# The Circuitry of Olfactory Projection Neurons in the Brain of the Honeybee, *Apis mellifera*

**DOI:** 10.3389/fnana.2016.00090

**Published:** 2016-09-29

**Authors:** Hanna Zwaka, Daniel Münch, Gisela Manz, Randolf Menzel, Jürgen Rybak

**Affiliations:** ^1^Institute of Neurobiology, Free University BerlinBerlin, Germany; ^2^Abteilung Genetik von Lernen und Gedächtnis, Leibniz Institut für NeurobiologieMagdeburg, Germany; ^3^Neurobiology, University of KonstanzKonstanz, Germany; ^4^Bernstein Center for Computational NeuroscienceBerlin, Germany; ^5^Department of Evolutionary Neuroethology, Max Planck Institute for Chemical EcologyJena, Germany

**Keywords:** projection neurons, honeybee, GABA, octopamine, olfaction, standard brain, antennal lobe tract

## Abstract

In the honeybee brain, two prominent tracts – the medial and the lateral antennal lobe tract – project from the primary olfactory center, the antennal lobes (ALs), to the central brain, the mushroom bodies (MBs), and the protocerebral lobe (PL). Intracellularly stained uniglomerular projection neurons were reconstructed, registered to the 3D honeybee standard brain atlas, and then used to derive the spatial properties and quantitative morphology of the neurons of both tracts. We evaluated putative synaptic contacts of projection neurons (PNs) using confocal microscopy. Analysis of the patterns of axon terminals revealed a domain-like innervation within the MB lip neuropil. PNs of the lateral tract arborized more sparsely within the lips and exhibited fewer synaptic boutons, while medial tract neurons occupied broader regions in the MB calyces and the PL. Our data show that uPNs from the medial and lateral tract innervate both the core and the cortex of the ipsilateral MB lip but differ in their innervation patterns in these regions. In the mushroombody neuropil collar we found evidence for ALT boutons suggesting the collar as a multi modal input site including olfactory input similar to lip and basal ring. In addition, our data support the conclusion drawn in previous studies that reciprocal synapses exist between PNs, octopaminergic-, and GABAergic cells in the MB calyces. For the first time, we found evidence for connections between both tracts within the AL.

## Introduction

Honeybees can discriminate a large range of odors (von Frisch, 1915; Laska et al., 1999; Guerrieri et al., 2005). Correlates of odor coding have been studied at the neural level at several stages of odor processing in ORN (Akers and Getz, 1993), in the AL (Joerges et al., 1997; Galizia et al., 1999b; Galizia and Menzel, 2000), in olfactory PNs of the ALT (Abel et al., 2001; Müller et al., 2002; Sachse and Galizia, 2002; Krofczik et al., 2008; Brill et al., 2013; Carcaud et al., 2015) and in the MB (Szyszka et al., 2005, 2008; Galizia and Rössler, 2010; Haehnel and Menzel, 2010; Froese et al., 2014). Axons of ORNs project from the antennae to the AL via four antennal tracts, T1–T4 (Suzuki, 1975; Mobbs, 1982; Abel et al., 2001). Here, approximately 160 glomeruli serve as the functional and anatomical units of the AL. These glomeruli can be allocated to clusters by the innervation of the four antennal tracts. As each glomerular cluster receives input from one antennal tract, they are termed the T1–T4 clusters (Flanagan and Mercer, 1989; Galizia et al., 1999a; Abel et al., 2001; Kelber et al., 2006) or A, B, C, and D glomeruli (Galizia et al., 1999a). PNs project via five AL output tracts, the median- (m-ALT), the lateral (l-ALT)- and three medio-lateral tracts (ml-ALTs) from the AL to the central brain (Bicker, 1993; Abel et al., 2001; Müller et al., 2002; Kirschner et al., 2006). About 900 PNs of the m-ALT and the l-ALT, receive input from single AL glomeruli and are therefore referred to as uPNs (Rybak and Eichmueller, 1993; Rybak, 2012). Multiglomerular PNs project via the three medio-lateral tracts from the AL to the PL without invading the MB. Contrarily, the uPNs project via the m-ALT and the l-ALT to the calyces of the MBs and to the LH. In uPNs, the neuronal paths targeting the central brain areas differ: m-ALT neurons leave the AL dorsally, send axons to the ipsilateral MB and then project to the LH and the LPL. In contrast, l-ALT neurons send axons to the LH first and subsequently project to the ipsilateral MB. Additionally, they originate within different glomerular clusters: m-ALT neurons receive input from the T2–T4 clusters of glomeruli, whereas l-ALT neurons get input primarily from T1 glomeruli and to some extent from the T2 and T3 glomerular clusters (Mobbs, 1982; Abel et al., 2001).

Physiologically, these dual olfactory pathways differ in their response latencies and their olfactory tuning properties, thus suggesting a functional differentiation (Müller et al., 2002; Menzel and Manz, 2005; Krofczik et al., 2008; Yamagata et al., 2009; Carcaud et al., 2012). Although, much information about the response profiles of PN neurons has been gained from electrophysiological recordings as well as calcium imaging studies (e.g., Abel et al., 2001; Müller et al., 2002; Krofczik et al., 2008; Denker et al., 2010; Brill et al., 2013; Carcaud et al., 2015), the way this information is distributed within the MB and protocerebral intrinsic circuitry is still unknown. The two olfactory uPN pathways are known to target partially overlapping subregions of the lip neuropil and innervate the basal ring of the MB calyces (Abel et al., 2001; Gronenberg and Lopez-Riquelme, 2004). Since they differ in their response latencies and their olfactory tuning properties, one may suspect a separation of subregions according to function within the MB calyces. After conducting mass tract staining analyses Kirschner et al. (2006) suggested that the cortex of the calyx lip is innervated by m-ALT neurons whereas the core of the calyx lip is innervated predominantly by l-ALT neurons. Similarly, in the LH, uPNs of the two tracts occupy different zones (Abel et al., 2001; Kirschner et al., 2006; Roussel et al., 2014). However, little is known about the anatomical differences on the level of individual uPN neurons of the two tracts (m-ALT and l-ALT), in particular with respect to their arborization patterns in the MB calyces.

Both types of neurons, m-ALT and l-ALT, display morphological specializations, and boutons along and on their axonal terminals in the lip and basal ring region of the MB calyx and in the LPL, including the LH. These varicosities are predominantly presynaptic specializations (Mobbs, 1982; Gronenberg, 1986; Rybak and Menzel, 1993; Grünewald, 1999b; Ganeshina and Menzel, 2001), indicating that the MB lip and basal ring receive olfactory input via uPNs. Thus the MBs comprise second order neuropils of the olfactory pathway. Moreover they are known to be involved in learning and memory formation in insects (Heisenberg, 2003; Menzel, 2012) as well as in forms of learning such as configural discriminations (Devaud et al., 2015). Two ventral unpaired median neurons of the maxillary and mandibular neuromere of the SEZ, VUMmx1 and VUMmd1, also project to both ALs, the PL, and invade the lip and basal ring regions of the MB calyces (Hammer, 1993; Schröter et al., 2007). The putative octopaminergic neuron, VUMmx1 (Kreissl et al., 1994; Sinakevitch et al., 2005, 2011; Schröter et al., 2007) encodes the reward stimulus in odor learning and acts as a modulatory neuron of the olfactory pathway (Hammer, 1993). Octopamine (OA) is a well-established neuromodulatory transmitter of the insect brain and plays an important role during associative and non-associative learning in the honeybee (Hammer and Menzel, 1995, 1998; Farooqui, 2007). Almost all neuropils of the subesophageal and supraoesophageal ganglion are innervated by octopaminergic neurons (Kreissl et al., 1994; Schröter et al., 2007) and the AL and the MB calyces are in part formed by VUM neurons (Kreissl et al., 1994; Rybak et al., 2001; Sinakevitch et al., 2005, 2011; Schröter et al., 2007). However, the neuronal odor-processing network in the MB is only partially understood. Octopaminergic neurons in the calyces probably target GABA-ergic neurons (Sinakevitch et al., 2011). GABA-IR profiles in the MB calyces belong to the A3 group of MB extrinsic neurons and form a recurrent pathway, via the PCT (Rybak and Menzel, 1993; Grünewald, 1999a). PCT neurons appear to receive inputs from the VUM neurons at the level of the MB lip region indicating that olfactory MB input via the ALTs is modulated by this putative inhibitory pathway (Ganeshina and Menzel, 2001; Sinakevitch et al., 2011). The GABAergic recurrent neurons connect the MB output region with all three calycal subregions (lip, collar, and basal ring), suggesting specific inhibitory feedback from the MB output to its input (Mobbs, 1982; Rybak and Menzel, 1993; Grünewald, 1999a).

Here, we investigate anatomical differences between uniglomerular m-ALT and l-ALT neurons at the single cell level using intracellular staining, 3D reconstruction and image registration to the honeybee standard brain atlas to derive the spatial relations and quantitative morphology of the neurons of both tracts on a single cell level. We compare our results to previously published data from uPN mass staining. In addition, we evaluate the putative synaptic connections between uPNs, GABAergic PCT neurons, and octopaminergic cells at the single cell level to gain a better understanding of the neuronal network in the MB calyces of the honeybee brain.

## Materials and Methods

### Animals

During the summer months, worker honeybees (*Apis mellifera carnica*) were caught at the hive entrance. In winter, pollen and sugar-water foraging animals flying in an indoor flight room were collected at the feeding sites. The bees were immobilized by cooling, mounted in Plexiglas tubes, and kept in a high humidity chamber. At night, the bees were held in a humid chamber in the dark at 20°C.

### Iontophoretic Staining

Dissection began with fixing the antennae to the head capsule with Eicosan, a low temperature melting wax (Sigma-Aldrich, München, Germany). The mandibular muscles were cut and the esophagus was fixed by pulling it out through a small window, followed by cutting the esophagus between the mandibles and the antennal joints, and then waxing it onto the clypeus. The head capsule was opened and the glands removed. A chloride-coated silver hook (ground electrode) was placed under the AL or into the compound eye. Brain movement was reduced by gently squeezing the abdomen with a stopper (boxing wax sticks, Kerr Corp., Washington, DC, USA). Immediately before inserting the tip of the microelectrode into the brain, the tracheae and the neural sheet were carefully removed above one AL. Bee Ringer solution [NaCl (130 mM), KCl (6 mM), MgCl_2_ (4 mM), CaCl_2_ (5 mM), glucose (25 mM), sucrose (170 mM) adjusted to pH 6.7 using diluted HCL] was applied when necessary to avoid drying of the brain surface. Microelectrodes were pulled with a horizontal laser puller (P 2000, Sutter Instruments, Corp., Novato, CA, USA) using borosilicate glass capillaries with a filament (Hilgenberg GmbH, Malsfeld, Germany) with an outer diameter of 1 mm, a length of 75 mm and a wall thickness of 0.21 mm. The tip of the electrode was filled with a 5% (w/v) Neurobiotin solution (Neurobiotin tracer, Vector Laboratories, Inc., Burlingame, CA, USA) or a 5% (w/v) tetramethylrodamine-biotin solution (TMR, 3000 MW, Microruby, MoBiTec, Göttingen, Germany) in 0.2 and 1 M potassium acetate, respectively. Resistance varied between 90 and 300 MΩ, depending on the backup solution. The electrodes were positioned and moved with 3D stepping motors (mini 25, Luigs & Neumann GmbH, Ratingen, Germany). After detecting intracellularly recorded spikes, the neurons were filled by injecting 1–4 nA depolarizing current for 10–20 min. The dye diffused overnight in the living animal held in a humidity chamber at 20°C. Additionally, mass fills of ALT neurons were performed. The broken tips of glass capillaries were loaded with TMR crystals and inserted into the dorso-posterior region at a location where both ALT tracts leave the AL-exit. The dye was allowed to dissolve and was taken up by injured neurons for several seconds. Afterward, the brains were rinsed in Ringer solution, the head capsule closed with Vaseline, and the animals were kept in a moist chamber overnight prior to further processing.

### Histochemistry

Brains were fixed with 4% (w/v) paraformaldehyde (PFA, Roth, Karlsruhe, Germany) solution and 0.4% (w/v) Lucifer yellow for enhanced background (Lucifer Yellow CH dilithium salt, Sigma-Aldrich, München, Germany) in phosphate buffered saline [PBS; NaCl (37 mM), KCl (2.7 mM), Na_2_HPo_4_ (8 mM), KH_2_Po_4_ (1.4 mM), adjusted to pH 6.7 using diluted HCL] or a mixture of 1.3% (w/v) PFA and 0.7% (v/v) glutaraldehyde (GA, Sigma-Aldrich, München, Germany) in PBS for at least 4 h. Subsequently, the brains were washed three times in PBS for 10 min each, dehydrated in ascending ethanolic solution series [50, 70, 90, 99, and 100% (v/v), each for 10 min], cleared in methylsalicylate (Roth, Karlsruhe, Germany) for 10 min, and mounted on a special object slide (a metal plate of 0.5 mm thickness with a central hole and cover slips on both sides) in methylsalicylate. Additionally, brains stained with Neurobiotin were permeabilized for 2 h in PBS containing 0.5 or 1% (v/v) Triton X (PBS-TX, Sigma-Aldrich, München, Germany) and incubated overnight at 4°C in 0.05% (w/v) indocarbocyanine (Cy3) or indodicarbocyanine (Cy5) coupled streptavidin (Dianova, Hamburg, Germany) 1:100 in PBS-TX containing 0.1% (w/v) sodium chloride (NaCl) for enhancement. The following day, the brains were washed at room temperature several times (for at least 2.5 h) in PBS, dehydrated and mounted as described above.

### Antibody Staining

Preparations with intracellularly stained neurons and mass fill ALT staining were treated with antibody staining against octopamine (mAb-oA1, mouse, Jena Bioscience, Jena, Germany), GABA (A2052 rabbit, Sigma-Aldrich, München, Germany), and/or GAD (Anti-GAD67, clone 1G10.2, MAB 5406 mouse, Millipore, Billerica, MA, USA).

### Octopamine Antibody Staining

Antibody staining against the neurotransmitter octopamine was performed with a monoclonal antibody. First, selected whole mount brains stored in methysalicylate were treated with a descending ethanolic solution series [100, 99, 90, 70, and 50% (v/v) each for 10 min]. Subsequently, the brains were washed three times in PBS for 10 min each. They were then treated for 30 min in 0.5% (v/v) PBS-TX and embedded in 5% (w/v) Agar (Amresco, low gelling temperature agarose) for vibratome sectioning. Brain slices (50–70 μm thick) were cut with a vibratome (VT 1000 S, Leica Microsystems, Wetzlar, Germany).

Slices were treated for 10 min with sodium borohydride 1% (w/v) (Sigma-Aldrich, München, Germany) in PBS to reduce fixative-induced auto fluorescence, washed in PBS for 15 min, and then for 30 min in 0.5% (v/v) PBS TX. Unspecific binding of antibodies was inhibited via blocking with 10% (w/v) normal goat serum (NGS, Linaris, Wertheim-Bettingen, Germany) and 1% (w/v) bovine serum albumin (BSA, Applichem, Gatersleben, Germany) in 0.5% (v/v) PBS TX for 1 h at room temperature. The primary antibody was applied at 1:1000 in 0.5% (v/v) PBS TX with 10% (w/v) NGS and 1% (w/v) BSA. 5 μl aqueous sodium azide solution was added to prevent fungal infection. The slices were incubated in the solution for 2 days at 4°C in the dark and then washed six times for 30 min in 0.5% (v/v) PBS TX. The secondary antibody goat-anti-mouse conjugated with the fluorophore carbocyanine (Cy2), Cy3 or Cy5 (depending on the former treatment, Dianova, Hamburg, Germany) was added at 1:200 or 1:100 with 5 μl aqueous sodium azide solution and incubated over night at room temperature. After washing the slices four times for 30 min in PBS, brains were washed in 1:3, 1:1, and 3:1 mixture glycerol/distilled water and then mounted in glycerin 3:1 in distilled water.

If more than one antibody (octopamine, GABA and/or GAD) was applied, the procedure was done as described above for the octopamine antibody staining. For whole mount antibody staining a treatment with 0.1% (w/v) collagenase-dispase, 0.1% (w/v) hyaluronidase (Sigma-Aldrich, München, Germany) in PBS for 30 min at 36°C. In addition, the brains were incubated for 20 min in sodium borohydride 1% (w/v) in PBS. Finally, the brains were dehydrated again as described before and mounted in methylsalicylate.

### GABA and GAD Antibody Staining

A primary polyclonal antibody against GABA was applied to show the presence of the inhibitory neurotransmitter GABA. In addition, antibody staining against GAD was performed. First, selected whole mount brains stored in methysalicylate were treated with a descending ethanolic solution series [100, 99, 90, 70 and 50% (v/v), each for 10 min]. Subsequently, the brains were washed three times in PBS for 10 min each. Afterward, they were treated for 30 min in 1% (v/v) PBS-TX and embedded in 5% (w/v) Agar for vibratome sectioning. Brain slices (50–70 μm thick) were cut with a vibratome.

Unspecific binding of antibodies was inhibited via blocking with 10% (w/v) NGS and 1% (w/v) BSA (only in GAD antibody staining) in 0.5% (v/v) PBS TX for 1 h at room temperature. The primary antibody was applied at 1:1000 in 0.5% (v/v) PBS TX with 10% (w/v) NGS and 1% (w/v) BSA (only in GAD antibody staining). 5 μl aqueous sodium azide solution was added to prevent fungal infection. The GABA primary antibody was incubated for 5 days, the primary antibody GAD for 2 days at 4°C in the dark and afterward washed six times for 30 min in 0.5% (v/v) PBS TX. The secondary antibody goat-anti-rabbit or goat-anti-mouse conjugated with the fluorophore carbocyanine (Cy2), Cy3 or Cy5 (depending on the former treatment) was added at 1:200 or 1:100 with 5 μl sodium azide solution and incubated over night at room temperature. After washing the slices four times for 30 min in PBS, brains were washed in 1:3, 1:1, and 3:1 mixture glycerol/distilled water and then mounted in glycerin 3:1 in distilled water.

### Confocal Imaging

Confocal image stacks of the whole brains or brain slices were acquired using a confocal laser scanning microscope (Leica TCS SP2, Wetzlar, Germany), using either a 10, 40, or 64x (NA: 0.4) IMM lens objective or a 20x (NA: 0.5) water lens objective. Sections were scanned at a resolution of 1024 × 1024 voxels each, and a voxel size of 0.61 μm × 0.61 μm × 1.3 μm, 0.73 μm × 0.73 μm × 1.1 μm or for distance mapping 0.23 × 0.23 × 1 and a step size of 0.1 μm. A 488 nm laser line was used for the neuropil stained tissue if Lucifer Yellow (0.4%, Lucifer Yellow CH dilithium salt, Sigma-Aldrich, München, Germany) or glutaraldehyde (GA, Sigma-Aldrich, München, Germany) was added and at 633 nm for the stained neuron. Immunostained preparations were scanned at 488, 543, or 633 nm depending on the used fluorophore. Linear intensity compensation was applied to adjust differences in brightness depending on scanning depth.

### Digital Reconstruction and Registration

All 3D reconstructions were done with Amira (Version 4.1., Mercury Comp, San Diego, CA, USA). Reconstruction of the PN morphology was done using a custom module (Evers et al., 2004; Schmitt et al., 2004).

First, the digitized neuron image was traced applying the snake algorithm to reconstruct a SkeletonGraph (Evers et al., 2004). In order to register the reconstructed neuron into the standard brain atlas of the honeybee (HSB^1^; Brandt et al., 2005; Rybak, 2012), the neuropils of the respective brain compartments (PL, MBs with its parts α-lobe, β-lobe, peduncle, and the calyces with lip, collar, and basal ring) were segmented using the Amira label field function. Following the protocol from Rybak et al. (2010) segmented brain neuropils were then affine registered onto HSB by applying linear transformations with respect to brain size and orientation. Afterward, an elastic registration algorithm was applied in order to perform local deformations for accurate spatial alignment to the HSB. The transformation data from these two registration procedures were then applied to the Skeleton Graphs of the reconstructed neurons that were thus visualized and spatially compared in the virtual space of the HSB.

Distance mapping between the reconstructed neuron and the neighboring tissue stained with the antibodies against GABA and/or octopamine was performed in Amira 4.1 applying a procedure as described in Evers et al. (2004). A false color scale was mapped on the surface of a reconstructed neuron’s dendrite, indicating the intensity of the fluorescence signal of surrounding antibody staining at a distance of 0.3 μm. Thus, warm colors (red) indicate high antibody staining intensity at a distance of 0.3 μm, which equals the distance from a vesicle pool to the post-synaptic site of a cell (Sätzler et al., 2002) thus indicating putative synapses.

### Image and Data Processing

Images were acquired using the Amira export function and, if necessary, adjusted in size with Adobe R Photoshop R Elements 2.0 (Adobe Systems, Inc., San Jose, CA, USA). Calculations were performed and all histograms computed using Calc 2.3.1 (Sun Microsystems, Inc., Santa Clara, CA, USA).

### Data Evaluation of Bouton Distribution

Neuropil labels of the MB were mapped onto SkeletonGraphs of neuronal arbors and its synaptic bouton so as to give every vertex of the SkeletonGraph an ID according to the neuropil it resides in. Information on SkeletonGraphs was exported to Open Office Calc (The Apache Software Foundation, Forest Hill, MD, USA) as a spreadsheet using the built-in Skeleton Stats module and with this data a bouton’s association to a neuropil could be extracted. If the stained cell was not eligible for reconstruction, boutons were counted in the original confocal images.

### Segmentation of the Lip Neuropil of the Mushroom Body Calyces

Surface models of the lips were generated based on the segmented lip label field and then manually dissected into subzones using Amira’s built-in path editor. The EvaluateBoutons module by Anja Kuss from the Zuse Institute Berlin was used to calculate a bouton’s association to one of the lip zones created and data was exported to Open Office Calc spreadsheet for further analysis. The lip zones applied are illustrated in **Figure 2D**.

### Bouton Distribution in the Lip Calyces

A centerline for each lip surface model was calculated using ZIB-Amira software. The EvaluateBoutons module was used to calculate two vectors for each bouton, its shortest distance toward the centerline and to the lip’s surface. With the lengths of these two vectors a relative distance from the centerline was calculated for every bouton according to the following equation: distance centerline/(distance centerline + distance surface) = relative distance from centerline.

### Statistics

For statistical analysis of multiple comparison, a Mann–Whitney *U*-test or a Wilcoxon matched pairs test was performed (Statistica version 8.0, StatSoft, Inc., Tulsa, OK, USA). Differences were considered statistically significant if *p* ≤ 0.05; if necessary, Holm–Bonferroni corrected *p*-values for multiple comparison were noted. Throughout this work, we use the term ‘significant’ as a description for a statistically significant difference. For normally distributed data, a repeated measurement ANOVA was used to analyze the interaction between two variables. For statistical comparison in **Figure 2H** values were log-transformed [T(x) = ln (1+x)]. All tests were performed using Statistica (Statistica version 8.0, StatSoft, Inc., Tulsa, OK, USA).

### Terminology

The terms for describing structural components of the honeybee brain have been adapted according to the nomenclature system of the Insect Brain Name Working Group (Ito et al., 2014). For identification and naming of glomeruli the honeybee AL atlas was used (Galizia et al., 1999a). Neurons of the ALTs were named according to their origin in the AL sub-regions (regions T1–T4) and their specific innervation of a glomerulus (groups A–D). The relative positions of brain neuropils are given according to the body axis.

## Results

**FIGURE 1:**
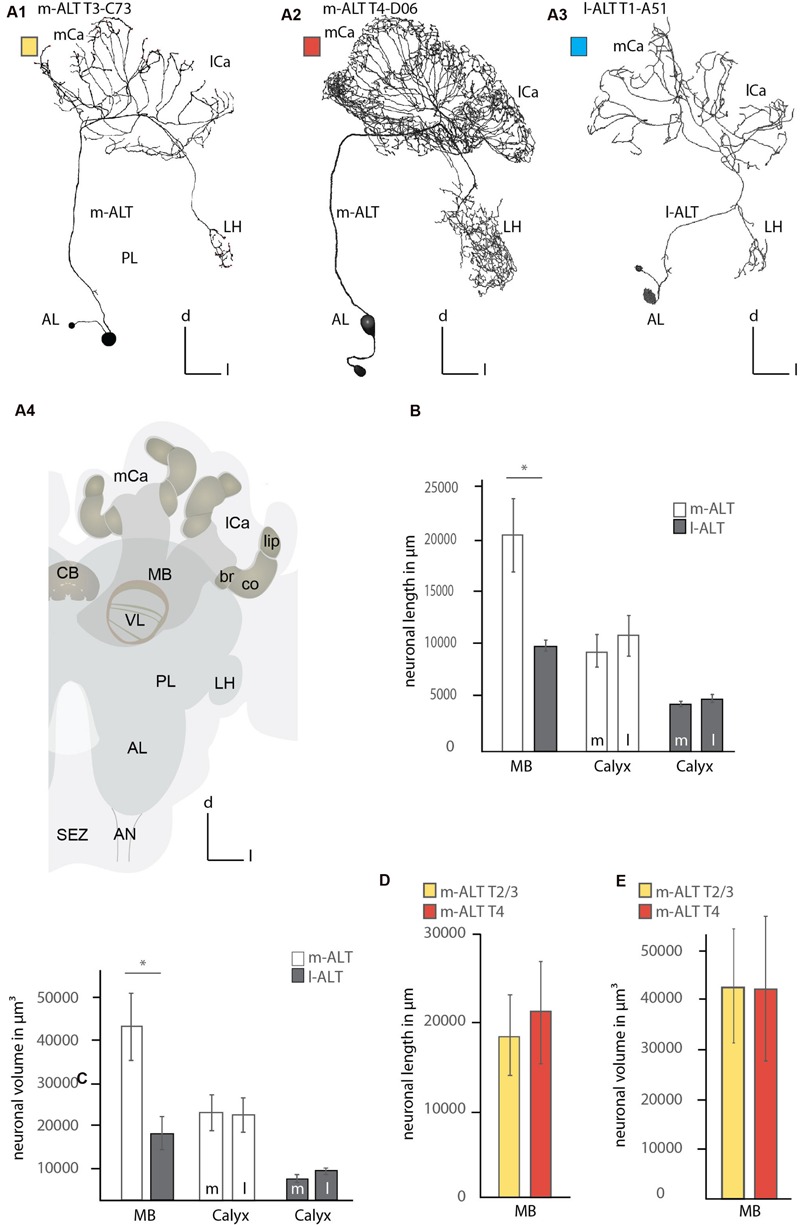
**Comparison of the innervation pattern and neuronal morphology of m-ALT and l-ALT neurons. (A1)** An m-ALT neuron of the sensory tract T3 cluster (glomerulus C73, T3-C73) with distinct, sparse domains in lip and basal ring of the MB calyces (mCa, lCa) with protocerebral arborizations restricted to the LH. **(A2)** An m-ALT neuron from the T4 cluster (glomerulus D06, T4-D06) arborizes over a large protocerebral area including the LH and forms extensive arborizations bearing a high number of boutons and overlapping domains in the lip of the mCa and lCa. **(A3)** An l-ALT neuron of the T1 cluster (glomerulus A5, T1-A51) with a similar morphology compared to m-ALT neuron shown in **(A1)**. It terminates in the outer part of the LH. **(A4)** A schematic of the major olfactory neuropils in the honeybee brain. The MB calyces (mCa, lCa) are composed of the lip (li), collar (co), and basal ring (br) neuropils. **(B)** Comparison of the neuronal length of m-ALT (white bars) and l-ALT neurons (gray bars) in both MB calyces (MB) and for the lateral (l) and medial (m) calyces separately. m-ALT neurons (T2–T4 glomerular cluster) have significantly longer arborizations in the MB-calyx than l-ALT neurons (Mann–Whitney *U*-test, *U* = 1, *z* = 2.55, *p* = 0.01, Holm–Bonferroni corrected *p* = 0.02, N_m-ALT_ = 6, N_l-ALT_ = 5). The neuronal length for either m-ALT or l-ALT neurons, respectively, does not differ comparing their innervation of the medial and the lateral calyx, respectively (N_m-ALT_ = 6, Wilcoxon matched pairs test, *z* = 2.20, *p* = 0.027, Holm–Bonferroni corrected *p* = 0.081, N_l-ALT_ = 5, Wilcoxon matched pairs test, *z* = 1.48, *p* = 0.14). **(C)** Comparison of the volume of axonal arborizations in the MB calyces and separately for the lateral and medial calyces for m-ALT neurons and l-ALT neurons. m-ALT neurons occupy significantly more volume than l-ALT neurons in the MB calyces (Mann–Whitney *U*-test, *U* = 3, *z* = 2.19, *p* = 0.028, Holm–Bonferroni corrected *p* = 0.028, N_m-ALT_ = 6, N_l-ALT_ = 5). Medial, and lateral calyx, do not differ with respect of arborizations volume of m- or l-ALT neurons, respectively (N_m-ALT_ = 6, Wilcoxon matched pairs test, *z* = 0.10, *p* = 0.92, N_l-ALT_ = 5, Wilcoxon matched pairs test, *z* = 1.78, *p* = 0.07). **(D)** The neuronal length in the whole MB calyx of T2/3 (yellow bars, *N* = 3) and m-ALT from T4- (red bars, *N* = 3) clusters of the AL (see also, **A1** and **A2**). **(E)** The axonal volume in the MB calyces of m-ALT from T2/3 cluster (yellow bars, *N* = 3) and T4 cluster (red bars, *N* = 3) of the AL (compare also **A1** and **A2**). Asterisk indicate statistical differences (Mann–Whitney *U*-test, *p* < 0.05). Error bars indicate the standard error of mean (SEM). AN, antennal nerve; br, basal ring; CB, central body; co, collar; d, dorsal; l-ALT, lateral antennal lobe tract; PL, protocerebral lobe; l, lateral; lCa, lateral calyx; LH, lateral horn; m, medial; m-ALT, medial antennal lobe tract; MB, mushroom body; mCa, medial calyx; VL, vertical lobe of the mushroom body; SEZ, subesophageal zone.

The reconstruction of single-cell stained neurons enabled a detailed analysis of the innervation patterns of medial and lateral antennal lobe tract (m-ALT and l-ALT), neurons, or PNs in the central honeybee brain (**Figure 1**, A1–A4; Supplementary Figure S1). We evaluated a total of 12 uniglomerular ALT neurons, six from each tract. The m-ALT neurons innervated glomeruli from the T2, T3, and T4 clusters of the AL [B05, C73, C(x), D02, D06, D08], while the l-ALT neurons innervated glomeruli from the T1 and T2 clusters [A(x), A33, A44, A51, A60, and B02]. The identity of two glomeruli could not be determined unambiguously, nevertheless they could be assigned to a specific cluster, named A and C, respectively [A(x) = T1, C(x) = T3]. We refer to the uPNs, or if the tract is important for understanding m-ALT or l-ALT neurons.

### m-ALT and l-ALT Neurons Differed in their Innervation Patterns

**FIGURE 2:**
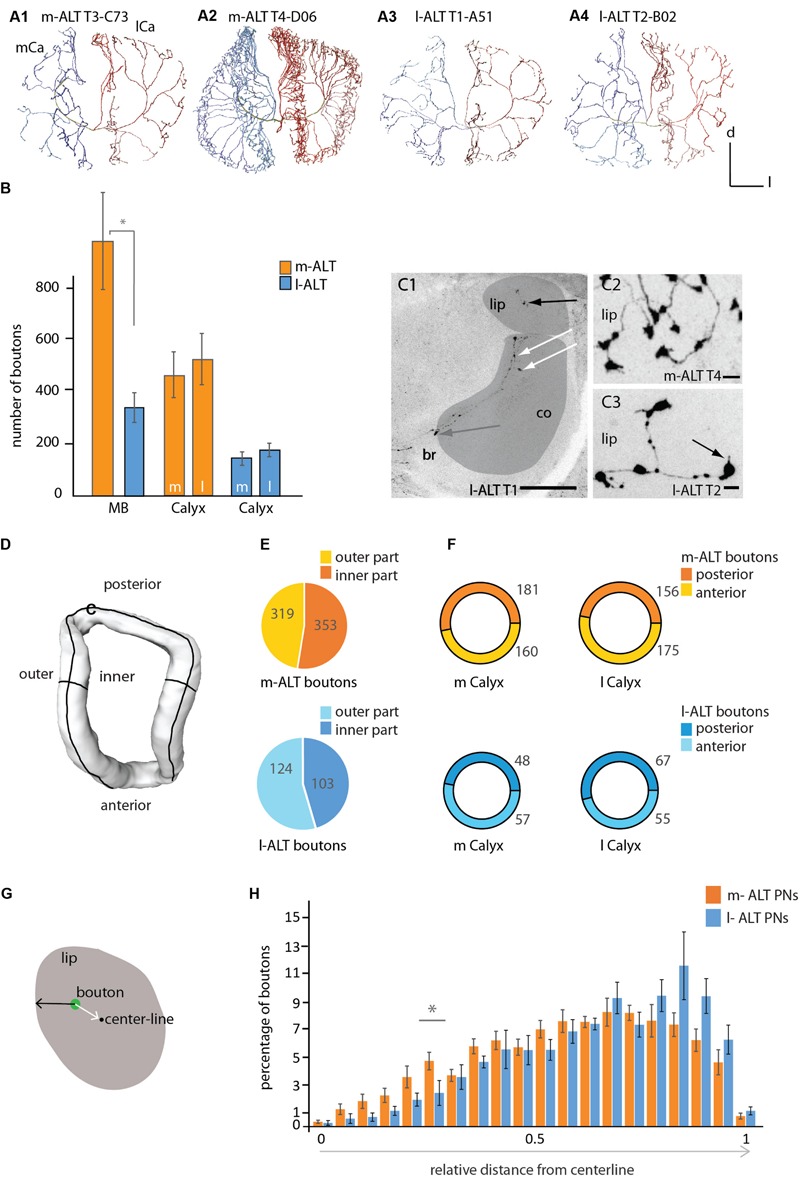
**Innervation patterns of projection neurons of the m-ALT and l-ALT and their numbers of boutons in the mushroom body calyces. (A1)** A sparse innervation of the MB calyx lip region by an m-ALT neuron from the T3 type (glomerulus C73) exhibiting distinct zones of axonal terminals. Each domain is separated from its neighboring area by a gap (see also **Figure 1**, **A1**). **(A2)** A m-ALT neuron from the T4 cluster (glomerulus D06) displays extensive arborizations with a high number of boutons and overlapping innervation zones in the lip. **(A3, A4)** Lip innervation of l-ALT neuron from the T1/2 cluster (**A3**: glomerulus A51; **A4**: glomerulus B02) innervated distinct domain-like lip segments. **(B)** Comparison of the total number of boutons (orange and blue bars) for the whole MB calyces (MB, left part of the graph) and of the total number of boutons in the lateral versus the medial calyces of m-ALTs (orange bars, *N* = 6) and l-ALTs (blue bars, *N* = 6) neurons (right part of the graph). Left: m- and l-ALT neurons differ significantly in their number of boutons (Mann–Whitney *U*-test, *U* = 2, *z* = -2.56, *p* = 0.01, Holm–Bonferroni corrected *p* = 0.03, N_m-ALT_ = 6, N_l-ALT_ = 6). Right: the number of boutons compared between both lateral-and median calyces of both neuronal populations do not differ significantly for m-ALT neurons (N_m-ALT_ = 6, Wilcoxon matched pairs test, *z* = 1.78, *p* = 0.074) and l-ALT neurons (N_l-ALT_ = 6, Wilcoxon matched pairs test, *z* = 2.20, *p* = 0.027, Holm–Bonferroni corrected *p* = 0.081). **(C1)** Frontal sections of the median calyx with an intracellularly stained l-ALT neuron exhibiting boutons in the lip (black arrow), in the collar (co, white arrows) and basal ring (br, gray arrow). Scale = 50 μm. Lip and collar marked in gray. **(C2)** Sections of the lip with a T4 m-ALT neuron forming *en passant* and terminal boutons. Scale = 5 μm. **(C3)** Sections of the lip with a T2 l-ALT neuron with *en passant* and terminal boutons with spiny extensions (black arrow). Note, the size differences between en-passant and terminal boutons. Scale = 5 μm. **(D)** Sketch of lip compartments (surface model) that were analyzed in **(E)**, inner and outer part and **(F)**, the posterior and anterior region of the lip. **(E)** Pie chart depicting percentage of boutons in the outer- (in yellow and light blue) and inner- (in orange and dark blue) lip for m-ALT (orange, *N* = 6) and l-ALT (blue, *N* = 6) neurons. Numbers give the mean value of boutons. m-ALT neurons have more boutons in the inner than in the outer lip part whereas l-ALT neurons display more in the outer part (not significantly; N_m-ALT_ = 6, inner: 353 ± 89, outer: 319 ± 31, Wilcoxon matched pairs test, *z* = 0.10, *p* = 0.92; N_l-ALT_ = 6, inner: 124 ± 21, outer: 103 ± 25, Wilcoxon matched pairs test, *z* = 0.31, *p* = 0.75). **(F)** Ring pie chart depicting percentage of boutons in the posterior (darker color) and anterior (lighter color) lip for m-ALT (orange, *N* = 6) and l-ALT (blue, *N* = 6) neurons, separately displayed for the medial (m Calyx) and lateral calyx (l Calyx). Numbers give the mean value of boutons. m-ALT neurons do form more boutons in the posterior than in the anterior part of the m Calyx and form more boutons in the anterior than in the posterior part of the lateral calyx (not significant), whereas l-ALT neurons boutons are arranged in the reverse manner (m-ALT: N_m-ALT_ = 6, m-posterior: 181 ± 39, m-anterior: 160 ± 32, Wilcoxon matched pairs test, *z* = 1.36, *p* = 0.17; l-posterior: 156 ± 31, l-anterior: 175 ± 38; Wilcoxon matched pairs test, *z* = 0.10, *p* = 0.92; l-ALT: N_l-ALT_ = 6, m-posterior: 48 ± 10, m-anterior: 57 ± 8, Wilcoxon matched pairs test, *z* = 1.6, *p* = 0.12; l-posterior: 67 ± 10, l-anterior: 55 ± 11, Wilcoxon matched pairs test, *z* = 0.1, *p* = 0.91). **(G)** Schematic cross section of the lip of the MB calyces showing the relative distance of a bouton (in green) from the centerline and surface of the lip neuropil. The number of boutons and its relative distance from the centerline is shown in **(H)**. **(H)** Histogram shows the distribution of boutons in the lip with regard to their location between the centerline and the surface for m-ALT PNs and l-ALT PNs, respectively. Boutons are binned in 20 classes of equal width distance from the centerline. Bouton quantity is displayed as percentage of the absolute bouton number of each neuron in the lateral and medial calyx. In the relative distribution we find a significant difference at 0.25–0.3 relative distance to centerline [rmANOVA *F*(19,266), 2,2340, *p* = 0.002; Tukey HSD *p* = 0.003 at 0.25–0.3, asterisk]. For absolute bouton number in every single cell please compare Supplementary Figure S4. Scale = 50 μm. AL, antennal lobe; br, basal ring; Ca, calyx; co, collar; d, dorsal; l, lateral; l-ALT, lateral antennal lobe tract; m, medial; m-ALT, medial antennal lobe tract; MB, mushroom body. Asterisk indicate statistical differences (Mann–Whitney *U*-test *p* < 0.05). Error bars indicate SEM.

M- and l-ALT neurons originating from different locations of the AL, do occupy similar but distinct neuropils of the central brain. M-ALT neurons from the T2 and T3 cluster of the AL, and l-ALT neurons of the T1 region showed sparse innervation in the PL restricted to the LH (**Figure 1**, A1, A3). Both groups occupy different zones. M- ALT neurons tend to arborize more centrally, whereas l-ALT terminals occupy the outer flanks of the LH (**Figure 1**, A1, A3; Supplementary Figure S1). In contrast, m-ALT innervating the T4 glomeruli arborized largely across a broad region of the lateral PL. In the MB calyces l-ALT and m-ALT neurons innervated all calycal subzones (lip, collar and basal ring, **Figure 1**, A4) but were most abundant in the lip region of the calyces. Here, l-ALT and one m-ALT (T3-C73) exhibited sparse innervation (**Figure 1**, A1, A3 and **Figure 2**, A1, A3–A4; Supplementary Figure S1), whereas m-ALT neurons of the T2/3 and T4 regions exhibited more innervations of the MB calyces (**Figure 1**, A2 and **Figure 2**, A2; Supplementary Figures S1 and S2).

Comparing the neuronal length of the axonal arborizations in the calyces within both MB calyces including its lip, basal ring, and collar sub-compartments showed that m-ALT neurons had a significantly longer neuronal length (sum of all axonal arborizations) compared to l-ALT neurons (**Figure 1B** left part, see Figure legend for statistical analysis). In the analysis every ALT branch entering the MB was taken into account with all its arborizations.

Additionally, we compared the axonal length of m-ALT and l-ALT neurons for the lateral versus the median calyx, respectively. We found, for neurons of both tracts, that their axonal length within the medial compared to the lateral calyx did not differ (**Figure 1B**, right part).

Additionally, we compared the axonal volume for all neurons. This also includes density and volume of boutons which can differ in number for distinct neuronal types and along the length of different neuronal arbors. Analyzing the axonal volume of the neurons in the MB calyces for m-ALT and l-ALT neurons showed that m-ALT neurons occupied significantly more volume than l-ALT neurons (**Figure 1C**, left). For both tracts the axonal volume between the medial and lateral calyx innervation did not differ (**Figure 1C**, right). Three of the six m-ALT neurons used in this study belonged to the T4 and three to the T2 and T3 AL glomerular clusters. Due to their different innervation in the PL we also compared length and volume for these two types of neurons in the MB calyces. The neurons belonging to the T4 tract were slightly longer, albeit not more voluminous than neurons from the T2 and T3 cluster (**Figures 1D,E**). We did not perform statistical analysis due to the small sample size of T2-3 and T4 m-ALTs, respectively.

### m-ALT and l-ALT Neurons Differed in Bouton Number and Distribution

Comparing the arborizations of uPNs in all calycal regions revealed different innervation patterns for m-ALT and l-ALT neurons. The individual uPN either reached the MB peduncle from its medial (m-ALT) or lateral (l-ALT) side and ran around the peduncle necks joining the IRT (Mobbs, 1982). They sent off collaterals toward the basal ring, collar and lip region of each MB calyx. Both neuron types formed terminal- and en-passant boutons in all calycal compartments (lip, collar and basal ring). Our data reveals that uPNs exhibit boutons in the inner rim of the calyx collar (**Figure 2**, C1).

One T2–T3 m-ALT neuron showed patterns with domain-like lip segments where they arborized without overlapping domains from branches deriving from neighboring axonal branches emanating from the IRT. The innervated areas were clearly separated (**Figure 1**, A1 and **Figure 2**, A1). In contrast, other m-ALT T2/3 PNs and all m-ALT neurons of the T4 glomerular cluster (type m-ALT T4) had extensive arborizations with a high number of boutons and overlapping axonal branches in the lip of the MB calyces (**Figure 2**, A2).

Innervation patterns of the l-ALT neurons were found to have a lower degree of arborization and less boutons in the calycal compartments and distinct axonal innervation zones in the lip (**Figure 1**, A3 and **Figure 2**, A3, A4). m-ALT neurons were equipped with significantly more boutons in the whole MB calyces including lip, basal ring and collar than l-ALT neurons (**Figure 2B**, for a comparison between m-ALT T2/3 and T4 see Supplementary Figure S3).

Comparing the lateral and the median calyx we found no difference in the number of boutons, neither for m-ALT neurons, nor for l-ALT neurons (**Figure 2B**).

In general, boutons occurred in three positions: along the neurite as *en passant* boutons, at branching points (**Figure 2**, C1 black arrow, C2, C3), and at the axon terminal (**Figure 2**, C3, black arrow). T2/3 m-ALT neurons had rounder boutons similar to those of l-ALTs. Big, rosette-like boutons expressed by T4 m-ALT neurons were found exclusively within the lip (**Figure 2**, C2, C3). Sometimes uPN boutons additionally formed spiny structures in the lip (**Figure 2**, C3 black arrow). l-ALT neurons displayed additional tiny boutons between the regular boutons. In the following, we restrict our analysis to the lip region of the calyces. To analyze the ALT-bouton distribution in the lip, we manually divided the lip region into anterior, posterior as well as inner- and outer parts and quantified the number of all boutons in these subdivisions (**Figures 2D–F** and Supplementary Data).

We found no difference in the number of uPN boutons when we compared their distribution in the posterior and anterior part of the lip for both m-ALT and l-ALT neurons (**Figure 2F**, for more details see also Supplementary Figure S3). Kirschner et al. (2006) suggested that m-ALT and l-ALT neurons innervate different areas of the MB lips. From mass staining of both tracts, they concluded that both neuron types innervate a central core but l-ALT neurons dominate this core part of the lip while the outer cortex of the lip is exclusively innervated by m-ALT neurons. Therefore, we performed a more detailed analysis of a potential morphological subdivision of the MB lips, of uPNs on the single cell level. Due to the lack of a clear definition of MB lip-zones we performed two different analyses: first, we counted the number of boutons in the inner- and outer part of the lip (**Figures 2D,E**) to test for a subdivision as indicated by a schematic overview provided by Kirschner et al. (2006). Second, we measured the distribution of boutons in the lip with regard to their location relative to the lip’s centerline (**Figures 2G,H**, see also Supplementary Figure S4) to test for a concentric subdivision of the lip via the m-ALT and l-ALT neurons, respectively. At the single cell level (**Figure 2E**) our data did not support Kirschner et al.’s (2006) study. For both tracts, we found no differences in the innervation pattern of the inner and outer parts of the lip (**Figure 2E**).

As m-ALT and l-ALT neurons differed in the number of boutons in the lip (**Figure 2B**), for our analysis of the distribution of boutons in core and cortex we considered the relative values instead of the absolute values. Therefore, all values were log-transformed [T(x) = ln(1+x)]. Our analysis of the concentric bouton segregation in twenty zones (**Figure 2H**) did not show any differentiation in core and cortex with respect to m-ALT and l-ALT innervation in the lip. Statistically, both bouton distributions did differ, however, a *post hoc* test reveals that only at 0.25 and 0.3 relative distance from centerline a significant difference in bouton number per zone for m-ALT and l-ALT occurs. However, the majority of all boutons from both the m-ALT and the l-ALT neurons was closer to the surface of the lip (that is: within a 0.5–1 relative distance centerline to surface where 0 represents centerline and 1 represents the neuropil surface, **Figure 2H**).

More precisely, the analysis of the distribution of the total number of boutons showed that the highest frequency was found around 0.7 relative distance from centerline for both cell types (0.65–0.85 for m-ALT and 0.65–0.90 for l-ALT). Interestingly, the difference in absolute number between the boutons of m-ALT and l-ALT neurons is most pronounced at this point (0.65–0.80 relative distance, **Figure 2H**). Here, m-ALT neuron boutons were in the majority (for single cell analysis please compare Supplementary Figure S4). Thus, this difference in absolute number of boutons might explain why Kirschner et al. (2006) suggested the difference in innervation by m-ALT and l-ALT neurons in lip cortex and core (**Figure 2H**).

### Anatomical Features of m-ALT Neurons in the Protocerebral Lobe and Antennal Lobes

**FIGURE 3:**
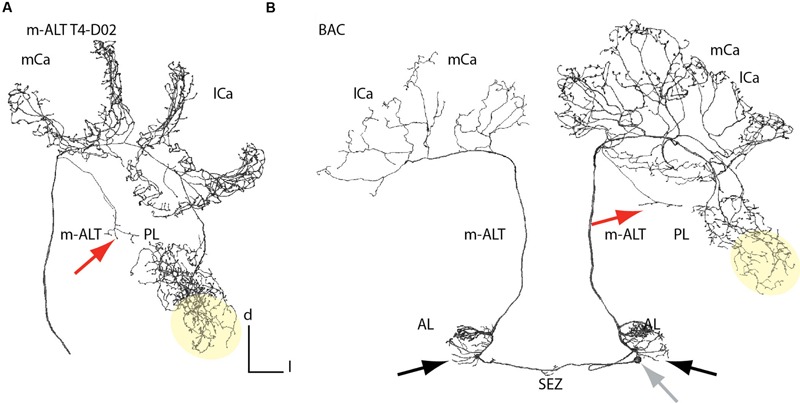
**Branching patterns of a unilateral and a bilateral m-ALT neurons connecting both hemispheres. (A)** Reconstruction of an m-ALT neuron of the T4 cluster (m-ALT T4-D02). **(B)** Reconstruction of a BAC neuron innervating glomeruli of the T4 cluster in each AL and exhibit extra-glomerular arborizations (black arrows). The soma is located in the SEZ at a depth of about 400 μm (gray arrow). Both neuron types exhibit a broad distribution of axon terminals in the lateral PL, including the LH (yellow area). In the PL a single axon was targeting the posterior PL (red arrows). The m-ALT T4-D02, and the BAC neuron, ipsilateral, exhibited overlapping domains with a high number of boutons in the mushroom body calyces. AL, antennal lobe; l-ALT, lateral antennal lobe tract; PL, protocerebral lobe; lCa, lateral calyx; m-ALT, medial antennal lobe tract; mCa, medial calyx; SEZ, subesophageal zone.

**FIGURE 4:**
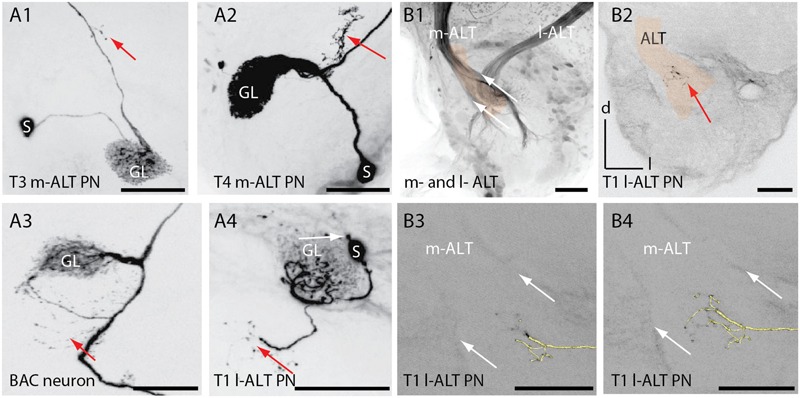
**Extra-glomerular arborization of ALT neurons in the antennal lobe. (A1)** Projection view of an T3 m-ALT neuron (GL: C73 glomerulus) with an axon projecting toward the exit of the antennal lobe (see **B2**). Red arrow points to boutons on fine dendrites emanating from the axon. **(A2)** Projection view of a T4 m-ALT neuron (GL: D02 glomerulus), forming collaterals outside the glomerulus (red arrow). **(A3)** Projection view of the BAC neuron with multiple fine arborizations in the AL emanating from the axon projecting to the T4-D05 glomerulus (red arrow). **(A4)** Projection view of a T1 l-ALT neuron with fine arborizations in the AL projecting from the innervated T1 glomerulus (A44, red arrow). The soma of this l-ALT neuron exhibits a nose like structure (white arrow). **(B1)** Frontal view of the AL at around 400 μm depth of the overlapping zone of m- and l-ALT leaving the AL (AL-exit, reddish label). **(B2)** Fine arborizations bearing boutons belonging to the T1 l-ALT neuron (see also, **A4**) projecting onto the m-ALT exit zone (red arrow). The reddish label marks the m-ALT. **(B3, B4)** Extra glomerular arborizations of an l-ALT fiber exhibiting terminal boutons at the m-ALT exit zone (white arrows, see: **B1**). In yellow: 3D reconstruction of the T1 l-ALT PN. Scale for **B1–B4**: 50 μm. AL, antennal lobe; l-ALT, lateral antennal lobe tract; GL, glomeruli; m-ALT, medial antennal lobe tract; PN, projection neuron; S, soma.

m-ALT neurons innervating the T4 cluster glomeruli displayed arborizations in the posterior PL outside the MB, which they sent off before entering the IRT They projected into an area posterior to the peduncle of the MB (**Figure 3A**, red arrow). These projections differed in length, but all of them displayed morphological specializations (boutons). None of the T2/3 m-ALT neurons or l-ALT neurons showed this morphological feature. Similar projections, we also found for a BAC (**Figure 4B**, red arrow). This AL output neuron connects both hemispheres of the brain. This class of bilateral AL output neurons was previously reported by Abel et al. (2001). Here, we showed that the BAC that innervates the same glomerulus (T4 cluster, D05) in both ALs, connected the ALs, and projected to both calyces. It displayed a large number of boutons and overlapping innervation zones in the lip and boutons in the basal ring on the ipsilateral side. It strongly innervated in the lateral PL, in addition to the arborizations in the posterior PL. The soma was located in the SEZ at a depth of about 400 μm (**Figure 3B**, gray arrow). The primary neurite bifurcated in the SEZ and sent two branches through the tritocerebrum, where it arborizes sparsely, and projected into each AL to the D05 glomeruli. Posterior to the D05 glomerulus, axons additionally formed several branches bearing boutons outside the D05 glomeruli of both ALs. One axon on each side left the ALs via the m-ALT first projecting to the calyces and then sending a branch into PL, similar to other T4 cluster m-ALT neurons. Innervation of the PL was only visible in the left brain hemisphere. The BAC neuron innervated both calyces with sparser innervation on the contralateral side, probably due incomplete staining. In both ALs, the neuron exhibited branches emanating from the axons running to the D05 glomeruli and to the m-ALT (**Figures 3B** and **4**, A3).

### Anatomical Features of m-ALT and l-ALT Neurons in the AL

Similar to the BAC neuron, m-ALT and l-ALT neurons formed extra-glomerular and bouton bearing arborizations in the AL (**Figure 4**, A1–A4, red arrows) as reported already by Müller et al. (2002). For the T1-A44 uPN we observed that these extraglomerular arbors projected into the area of the ALTs axon bundles shortly before both tracts leave the AL (AL overlapping zone at the AL exit, **Figure 4**, B1). A projection view of the fine arborizations within the AL revealed that fine dendrites deriving from the T1 l-ALT uPN axon project onto the m-ALT (**Figure 4**, A4 and B2, red arrows). This cell also exhibited an additional outgrowth structure on its soma (**Figure 4**, A4, white arrow).

### GABA Immunoreactive l-ALT Neuron

**FIGURE 5:**
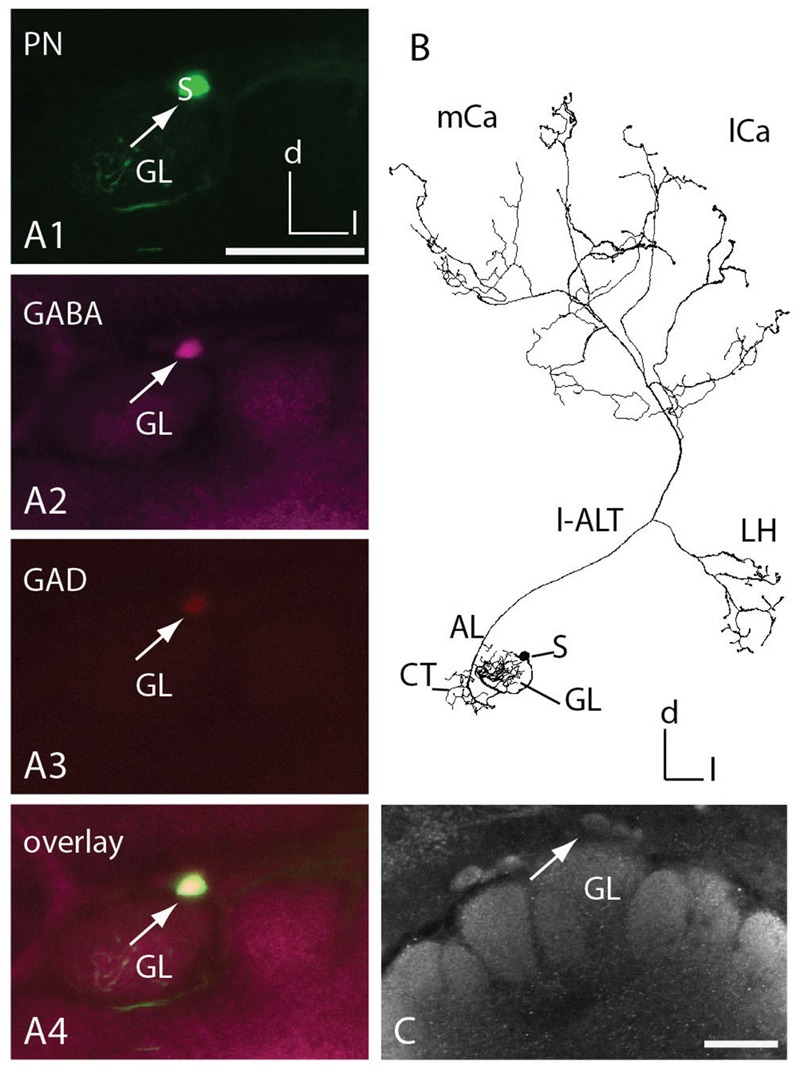
**T1 l-ALT neuron soma exhibits GABA- and glutamic acid decarboxylase (GAD) immunoreactivity. (A1–A4)** Co-localization of two neurotransmitters in an l-ALT PN. (**A1)** An intracellularly stained T1 l-ALT neuron (T1-A44, in green) originating in the dorsal somata cluster (s: soma, white arrow) of the left AL. Triple staining using anti-GABA (magenta, **A2**), anti-GAD (red, **A3**) reveals that the dye-filled l-ALT soma shows immunoreactivity against the enzyme GAD and the neurotransmitter GABA. **(A4)** Overlay image reveals co-localization of anti-GABA antibody staining, anti- GAD antibody staining and intracellularly marked neuron. Co-localization appears white to yellow. Images are collected at the same confocal settings of detector gain and laser intensity. **(B)** Full reconstruction of the T1 l-ALT-A44 with sparse innervations in the lip regions of the mushroom body calyces (mCa, lCa) and the LH of the PL. **(C)** Projection view of the dorsal antennal lobe stained with anti-GABA serum. White arrow points to a somata in a similar area as in **(A)**. AL, antennal lobe; Ca, calyx; CT, collaterals; d, dorsal; GL, glomerulus A44; l, lateral; l-ALT, lateral antennal lobe tract; LH, lateral horn; m, medial; S, soma. Scale = 50 μm.

Uniglomerular PNs in honeybees exhibit acetylcholine-like immunoreactivity (fibers of the m-ALT) and taurine-like immunoreactivity (fibers of the l-ALT; Schäfer et al., 1988; Kreissl and Bicker, 1989). Medio-lateral ALT neurons innervating several glomeruli in the AL run in a separate output tract to the central brain, and were found to contain GABA (Schäfer and Bicker, 1986; Bicker, 1999). Here, we report one l-ALT neuron, the T1-A44, that exhibited GABA-like and GAD-like immunoreactivity in its soma (**Figure 5A**) and adjacent primary neurite. It innervated glomerulus A44 in the T1 region at the ventral rind of the AL (**Figures 5A,B**). Arborizations of this neuron were similar to all known l-ALT neurons (**Figure 5B**, compare Supplementary Figure S1). In a different preparation GABAergic somata could be found in the same area (**Figure 5C**).

### Putative Synaptic Contacts in the MB

**FIGURE 6:**
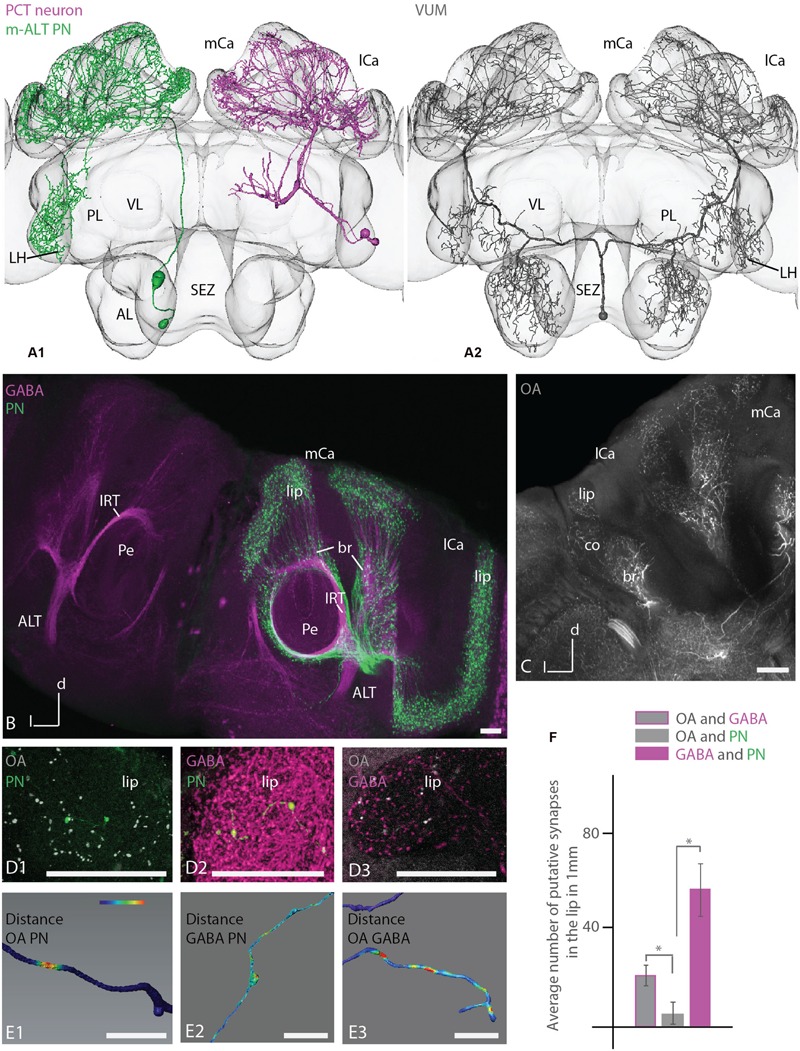
**Putative synaptic contacts of l-ALT neurons with antibodies staining against GABA and octopamine in the lip. (A) A1:** reconstruction of a T4 m-ALT neuron (T4-D06, in green), a GABAergic PCT neuron (deCamp, 2009; in magenta) and an octopaminergic VUMmx1 neuron (**A2**, in gray) registered to the Honeybee Standard Brain. All neurons have overlapping innervation areas in the MB calyces and the uPN and the VUMmx1 additionally in the LPL. **(B)** Projection view of anti-GABA antibody staining (magenta, left and right hemisphere of the brain) with adjacent ALT mass staining (in green, in the right hemisphere of the brain,) in the MB calyces (horizontal view). The GABA antibody staining is widely distributed in the calyces and closely adjoined by boutons formed by the ALTs (right hemisphere). Both neuron types enter the calyces via the IRT. **(C)** Overview of anti-octopamine antibody staining (in white) in the MB calycal region. The antibody staining is widely distributed in lip, collar and basal ring region, similar to the distribution of fibers of the VUMmx1 and VUMmd1 (see **A2** for VUMmx1). **(D1–D3**) Close-up on the lip region with anti-octopamine staining (**D1**, gray) and intracellularly stained uPN (**D1**, green), anti-GABA staining (**D2**, magenta and intracellularly stained uPN (**D2**, green), and anti-octopamine- (**D3**, gray) and anti-GABA staining (**D3**, magenta). (**E1–E3)** Distance mapping of closely neighbored neurons in the lip of the MB calyces. The neuron distance is false color coded with red coding a threshold distance of 0.3 μm indicating a putative synaptic contact. Warm colors represent close distances. **(E1**) Example of a reconstructed l-ALT neuron axon (PN) indicating its close distances to anti-octopamine fibers (OA). **(E2)** Example of an l-ALT neuron axon (PN) indicating putative contacts with surrounding anti-GABA neurons. **(E3**) Example of a single GABAergic fiber (GABA) indicating its close distance to OA fibers. **(F)** Average number of putative synapses between OAergic and GABAergic fibers, OA-ergic fibers and uPNs (PN) and GABA-ergic fibers and uPNs in the lip region, respectively. We find significantly more connections between GABAergic fibers and uPN than between OAergic fibers and uPNs (Mann–Whitney *U*-test, *U* = 0, *z* = 2.88, *p* = 0.003, N_OA/PN_ = 6, N_GABA/PN_ = 19). In addition, there are significantly more synaptic between OAergic fibers and GABAergic fibers than between OAergic fibers and uPNs (Mann–Whitney *U*-test, *U* = 0, *z* = 2.88, *p* = 0.004, N_OA/PN_ = 6, N_GABA/OA_ = 6). The difference between contacts of GABAergic profiles to uPNs and between contacts of GABAergic to OA profiles was not significant (Mann–Whitney *U*-test, *U* = 34, *z* = -1.46, *p* = 0.14, N_GABA/PN_ = 19, N_GABA/OA_ = 6). AL, antennal lobe; br, basal ring; co, collar; l-ALT, lateral antennal lobe tract; PL, protocerebral lobe; lCa, lateral calyx; m-ALT, medial antennal lobe tract; MB, mushroom body; mCa, medial calyx; OA, octopamine; Pe, peduncle of the mushroom body; VL, vertical lobe of the mushroom body; SEZ, subesophageal zone. Scale bar: 50 μm. Asterisk indicates statistical differences (Mann–Whitney *U*-test *p* < 0.05). Error bars indicate SEM. Neuron in **A1** reconstructed from a staining in deCamp (2009).

Reconstructed uPNs, GABAergic A3 neurons of the PCT and the putative octopaminergic VUMmx1 neuron showed overlapping innervation areas in the calyces documented by registering the respective neurons in the honeybee standard atlas (**Figure 6**, A1, A2). Additionally, uPNs and the VUMmx1 overlapped in the PL and ALs. Anti-GABA staining with adjacent ALT mass staining revealed similar innervation areas in the calyces (**Figure 6B**). GABA stained A3 neurons were widely distributed in the calyces, forming mostly bouton structures that are closely adjoined by axonal arborizations of the uPNs. Antibody staining against octopamine in the calycal region stained similar areas (**Figure 6C**). The OA staining was widely distributed in the lip, collar and basal ring region, analog to the distribution of the VUMmx1, VUMmd1 and other VUM neuron types (**Figure 6**, A2) (Rybak et al., 2001; Sinakevitch et al., 2005; Schröter et al., 2007) arborizing in these MB areas. The octopaminergic innervation in the calyx lip and basal ring was sparse, arising from two large fibers running in and parallel to the l-ALT, entering the calyces via the IRTs. This indicates that octopaminergic immunoreactivity in the lip and basal ring derives from the VUM neurons (Schröter et al., 2007; **Figure 6C**, compare Supplementary Figures S5).

A detailed investigation of the calyx lip region in different animals comparing intracellularly stained l-ALT neurons with anti-octopamine staining and anti-GABA staining, respectively (**Figure 6**, D1, D2), and anti-octopamine with anti-GABA staining (**Figure 6**, D3) revealed multiple close contacts between the cell populations (see also Supplementary Data). Using 3D neuronal reconstruction and automated co-localization analysis following the procedure by Evers et al. (2004) and Meseke et al. (2009) we identified putative synaptic contacts between these neuronal populations. A threshold distance of 0.3 μm was chosen to determine a synaptic contact. This length equals the distance from a vesicle pool to the post-synaptic site of a cell (Sätzler et al., 2002). We thus mapped close distances of OA- and GABA-IR fibers on uPN, and between OA-IR and GABA-IR fibers (**Figure 6**, D1–D3, E1–E3). A putative synaptic contact was mapped onto the surface model of the respective reconstructed neuron’s arborizations, indicating the intensity of surrounding antibody staining by a false colored scale. Warm colors display close distances (**Figure 6**, E1–E3). On average, a length of 1 mm of uPN arborizations in the lip contained 57 ± 10 SEM contacts to GABAergic profiles and 6 ± 3 SEM contacts to octopaminergic profiles (**Figure 6F**). Thus, contacts of PNs to GABAergic profiles were significantly more frequent than contacts of PNs to octopaminergic profiles (**Figures 6**, E1, E2 and **6F**). High anti-GABA antibody staining was also found around boutons of uPNs.

GABAergic and octopaminergic neurons contacted each other on average on 1 mm axonal length of their arborizations in the lip about 20 ± 2 SEM times (**Figure 6F**). This was significantly different from contacts between octopaminergic neurons and uPNs, whereas the difference between contacts of GABAergic profiles to uPNs and between contacts of GABAergic to octopaminergic profiles was not significant.

## Discussion

In the honeybee brain, two tracts – the lateral and the medial ALT – project from the primary olfactory center, the ALs, to the central brain, the MBs, and the PL (Bicker, 1993; Abel et al., 2001; Kirschner et al., 2006). We reconstructed individual intracellularly stained uPNs of both tracts, registered them to the 3D honeybee standard brain atlas (HSB), and compared the spatial relations and the morphology of the neuron types. Our 3D morphological analysis of these neurons revealed that lateral and medial uPNs (m-ALT and l-ALT neurons) differ in neuronal length and volume, as well as in number of boutons, that were indicative of putative presynaptic structures, in the central brain. Two types of m-ALT neurons (belonging to the T2/3 and the T4 cluster of glomeruli, respectively) can be distinguished, regarding their projection pattern in the PL. While the overall innervation patterns of m-ALT and l-ALT neurons were different, both displayed boutons in the core and cortex of the MB lip to equal amounts. This is in contrast to the results of previous mass staining (Kirschner et al., 2006). Moreover, a detailed morphological analysis of single uPNs in the calyces revealed boutons in the collar region, suggesting that the collar is a multimodal input site similar to the basal ring. Data from combination of single cells with antibody staining shows that reciprocal synapses exist between PNs, octopamine-, and GABA-ergic cells in the MB calyces. These results support conclusions from previous studies (Ganeshina and Menzel, 2001; Sinakevitch et al., 2011).

Additionally, for the first time, we found evidence for connections between both tracts at the level of the AL formed by extra-glomerular arborizations of ALTs at the common AL exit of both tracts. Non-glomerular axonal emanations of AL PNs were previously described by Müller et al. (2002).

### Anatomical Differences between m-ALT and l-ALT Neurons

We found differences between neurons of both ALT tracts regarding their innervation of the LH and the PL. m-ALTs tend to project more centrally in the LH compared to ALTs, Such an ALT-specific compartmentalization that was previously reported in the LH, suggests that different microcircuits are formed by both cell types (Abel et al., 2001; Kirschner et al., 2006). A zonation in the LH was shown with respect to processing odor valence (Jefferis et al., 2007; Galizia, 2014; Strutz et al., 2014) including distinct representations for different bee pheromones (Roussel et al., 2014). In *Drosophila melanogaster* the LH is segregated into zones according to pheromone versus fruit odor processing (Jefferis et al., 2007; Datta et al., 2008). In addition, T4 m-ALTs innervated broader areas in the PL and were not restricted to the LH as l-ALTs, thus resembling AL projections formed by the medio-lateral ALTs (Abel et al., 2001; Kirschner et al., 2006; Rybak, 2012).

In contrast to Kirschner et al. (2006), we could not confirm the segregation of olfactory input in the MB lip subcompartment. Although, our findings are based on a few single neurons, our data show unspecific innervation with regard to glomeruli origin of ALT neurons. Notably, the difference in neural density between l-and m-ALT fibers in the lip region, suggests that m-ALT neurons do contact a higher number of KC dendrites. In *Drosophila melanogaster*, KCs integrate olfactory input in a random fashion, variable across animals (Caron et al., 2013), as might be expected for brain structures involved in integration of multimodal sensory input and memory formation (Caron et al., 2013; reviewed in Menzel, 2014).

Protocerebral projection areas of the T4 m-ALT neurons of this study correspond to the broad band formed by two prominent ALT output areas, the lateral bridge and the triangle (Kirschner et al., 2006). Thus, direct uniglomerular input outside the LH from the AL to the PL seems to be restricted to the m-ALT cells innervating glomeruli of the T4 cluster and the BAC fibers (see also Abel et al., 2001). Also, these ALT neuron type overlaps with an ml-ALT neuron of multiglomerular origin in the AL (Rybak, 2012). These areas of the posterior PL receive multimodal input from visual fibers and mechanoreceptor neurons (Maronde, 1991).

Glomeruli of the T4 cluster are the biggest glomeruli in the AL (Flanagan and Mercer, 1989). In addition to olfactory cues they process input from other sensory modalities (e.g., taste, temperature, humidity; Abel et al., 2001; Kelber et al., 2006; Nishino et al., 2009). Recent work shows that this cluster might be homologs among different Hymenoptera species including ants, bees and hornets (Couto et al., 2016). Glomeruli in the T1–T3 clusters get only olfactory input (Abel et al., 2001; Müller et al., 2002; Kirschner et al., 2006). Brown et al. (2002) analyzed the ultrastructure of two glomeruli in *Apis mellifera* workers, A44 (T1 cluster) and D02 (T4 cluster). Both glomeruli showed an increase in volume when worker bees had left the hive and foraged. This outgrowth was accompanied by an increase in the number of synapses in the A44 but not in the D02 glomerulus (Brown et al., 2002). The authors suggest that most of the synaptic connections within this glomerulus are already made before adult emergence and hence it may play a role in tasks that are relevant for a honeybee worker from the beginning of its adult life, such as social interactions that take place via tactile or chemosensory cues. Thus, glomeruli of the T4 cluster might be specialized for compound stimuli received by the antennae, e.g., in the context of contact chemoreception of social interactions (Brown et al., 2002). Support for this interpretation comes from the recording and marking of a bilateral AL connecting (BAC, **Figure 3B**) neuron also described in Abel et al. (2001). The BAC neuron innervates the glomerulus D05 (T4 glomerular cluster) in both sides of the ALs and runs via the m-ALT toward the MBs and the PL. Here, we report that this cell (**Figure 3B**) innervates both ALs, projects to the ipsi- and contralateral MBs and has broad arborizations in the ipsilateral PL and MB (**Figure 3B**). Abel et al. (2001) reported that the cell responds to touching the antenna with beeswax contralateral to the soma. No response to the application of six different odors or mechanical stimulation was observed. The BAC neuron exhibiting bouton structures also in the dorsal lobe [or antenno-mechano motor center (AMMC)] suggests that this neuron might convey olfactory and mechanosensory information from the Johnston organ (Ai et al., 2009) or transfer chemosensory (Abel et al., 2001) information to the AMMC which takes part in multimodal sensory processing (Erber and Schildberger, 1980; Flanagan and Mercer, 1989; Maronde, 1991; Haupt, 2007). In contrast, in *Manduca sexta* a uniglomerular bilateral neuron with the soma lying in the SEZ that projects also into the calyces is odor responsive (Kanzaki et al., 1989). In *Drosophila melanogaster*, a bilateral neuron connecting the ALs with the MBs conveys information about CO_2_ (Bräcker et al., 2013; Lin et al., 2013).

m-ALT and l-ALTs have overlapping areas in the MB calyces but differ partly in their gross anatomy (Müller et al., 2002; Kirschner et al., 2006). Here, we showed that m-ALTs on average occupy broader regions and display more boutons whereas l-ALT neurons arborize more sparsely within the lip and basal ring and exhibit fewer synaptic boutons. They show sparse innervations within the MB lip areas separated by a gap from the neighboring branch, while m-ALT neurons especially from the T4 cluster lack distinct domains and show overlapping innervation zones in the lip (**Figure 2**). Thus, information from T4 glomeruli could be transferred to a broader population of KCs at the same time, whereas l-ALT neurons from T1 to T3 glomeruli may be more specific in targeting small populations of KCs.

Different modalities are known to be processed in the calycal subcompartments – the lip, collar, and basal ring. Olfactory uPNs predominantly innervate the lip, whereas visual PNs innervate the collar. The third compartment, the basal ring, gets input from both modalities (Mobbs, 1982; Gronenberg, 1986, 2001; Gronenberg and Lopez-Riquelme, 2004). For the first time, we describe uPNs exhibiting terminal boutons and *en passant* boutons in all three calycal compartments. Boutons in the distal collar zone derive from the ALT of the IRT (**Figure 2**, C1) and were not reported before. This bouton distribution indicates olfactory input into all three calycal compartments. The distal collar zone was previously described (Gronenberg, 2001; Strausfeld, 2002) as a gustatory input site (Schröter et al., 2007). As olfactory input is also conveyed to this zone, the collar can be described as a multimodal input site as reported for the basal ring (Gronenberg and Lopez-Riquelme, 2004).

Kirschner et al. (2006) reported differences in innervation patterns of the MB-calyces. They proposed that the lip cortex is only innervated by m-ALT neurons whereas the core of the lip is innervated predominantly by l-ALT neurons. Our single neuron analysis contradicts this notion. We divided the lip in an inner and outer part (**Figure 2D**) and calculated the number and distribution of boutons in these compartments. m-ALT and l-ALT neurons exhibit boutons in the outer part and in the inner part of the lip. An analysis for a concentric subdivision does also not indicate that the lip cortex is only innervated by m-ALT neurons whereas the core of the lip is innervated predominantly by l-ALT neurons (**Figure 2H**, see also Supplementary Figure S4). Contrarily, we find a significant difference in bouton distribution at 0.25–0.30 relative distance from centerline with presumably less l-ALT boutons. Given that m-ALT neurons, arborize massively in the lip region, in a massive staining as applied in the Kirschner study an innervation in the core by l-ALT neurons might be hidden. Only at the single cell level do these innervation patterns become obvious.

### Anatomical Features of m-ALT and l-ALT Neurons in the AL

Before exiting the AL, uPNs exhibit collaterals that project most likely to other AL neurons (Müller et al., 2002). For the first time we provide evidence of contact collaterals bearing boutons of an l-ALT neuron onto axons of the m-ALT at the AL exit, a region occupied by the outgoing ALT fiber tract (**Figure 4**). This indicates that information might be exchanged between the two tracts before they leave the ALs. This extra-glomerular network could play a role in learning-related plasticity in the olfactory pathway and/or may be related to spike synchronization (Müller et al., 2002). Evidence for extra-glomerular processing was also found in the case of the BAC neuron, which exhibited projections in the AL outside the D5 glomeruli (**Figure 3B**) and in the case of a bilateral neuron connecting the ipsilateral AL glomeruli with the contralateral non-glomerular AL neuropil (Rybak, 2012).

### GABAergic l-ALT Neuron

m-ALT neurons contain acetylcholinesterase, suggesting acetylcholine as the main transmitter of m-ALTs (Kreissl and Bicker, 1989). Soma clusters of l-ALT neurons at the anterior rind of the AL and fibers in the l-ALT were found to be taurine-like immunoreactive (Schäfer et al., 1988). However, PNs in *Drosophila melanogaster* are thought to be predominantly cholinergic (Yasuyama et al., 1995). In our study, one l-ALT soma and its adjacent arborizations were found to be GABAergic (**Figure 4**). GABA antibody staining repeatedly marked one neuron in the l-ALT (personal communication Irina Sinakevitch). So far, only fibers of the medio-lateral ALT displayed GABA immunoreactivity (Schäfer and Bicker, 1986). This result suggests inhibitory input into the MB via at least one uPN of the l-ALT.

### Putative Synaptic Contacts in the MB Calyces

The MBs are invaded by a prominent group of GABAergic cells, the PCT neurons (reviewed in Bicker, 1999). These feedback neurons, also named A3d and A3v, connect the MB output regions of the alpha and beta lobe with the lip, collar and basal ring (Gronenberg, 2001; Strausfeld, 2002). A3 neurons might be responsible for the inhibitory component of odor-evoked oscillatory responses of KCs (Laurent and Naraghi, 1994). Synaptic connections between A3 neurons and PNs were found in electron microscopy studies (Ganeshina and Menzel, 2001). Our results strengthen these findings. Close attachments as analyzed here by light microscopy are indicative for synapses between PNs and GABAergic A3 cells. Judging from the Ganeshina and Menzel (2001) study, these reciprocal contacts could work in both directions (Supplementary Figure S6). Some boutons analyzed in this study of the l-ALT exhibited no contact to anti-GABA stained fibers. This illustrates that most likely not all bouton varicosities of a particular uPN receive inhibitory input from PCT neurons.

The sustained activity of PNs in response to olfactory stimuli increases the sensitivity of the KCs to odors when correlated with sucrose stimulation (Szyszka et al., 2008). This modulatory effect is likely to be controlled by the VUMmx1 neuron that conveys appetitive reinforcement during reward learning of odors (Hammer, 1993; Hammer and Menzel, 1998). Szyszka et al. (2008) suggested that this putative octopaminergic neuron provides input to the KCs either directly or indirectly via the ALT presynaptic terminals. Here we present evidence that the VUMmx1 neuron also conveys the input to uPNs directly. A direct connection between KCs and octopaminergic neurons is supported by the finding that clawed KCs express the OA receptor AmOA1 in their axons, cell bodies, and dendrites (Sinakevitch et al., 2011).

In the MBs the OA receptor AmOA1 is also expressed on GABAergic feedback neurons, suggesting that octopamine modulates the activity of certain inhibitory circuits (Sinakevitch et al., 2011). This interpretation is supported by our findings of putative contacts between PCT neurons and the VUMmx1 (**Figure 6**, E3) and the results of Ganeshina and Menzel (2001). Therefore, it is likely that the octopaminergic VUMmx1 projects onto PCT neurons and modulates the inhibitory input onto both ALT boutons and KCs (Supplementary Figure S6). uPNs might thus receive inhibitory input via PCT neurons excited or modulated by the VUMmx1 during coincident sucrose stimulation. Calcium imaging studies revealed that the activity of KCs upon odor stimulation is intensified by GABA receptor blockage (Froese et al., 2014).

### Summary of the Anatomy of m-ALT and l-ALT Neurons

**FIGURE 7:**
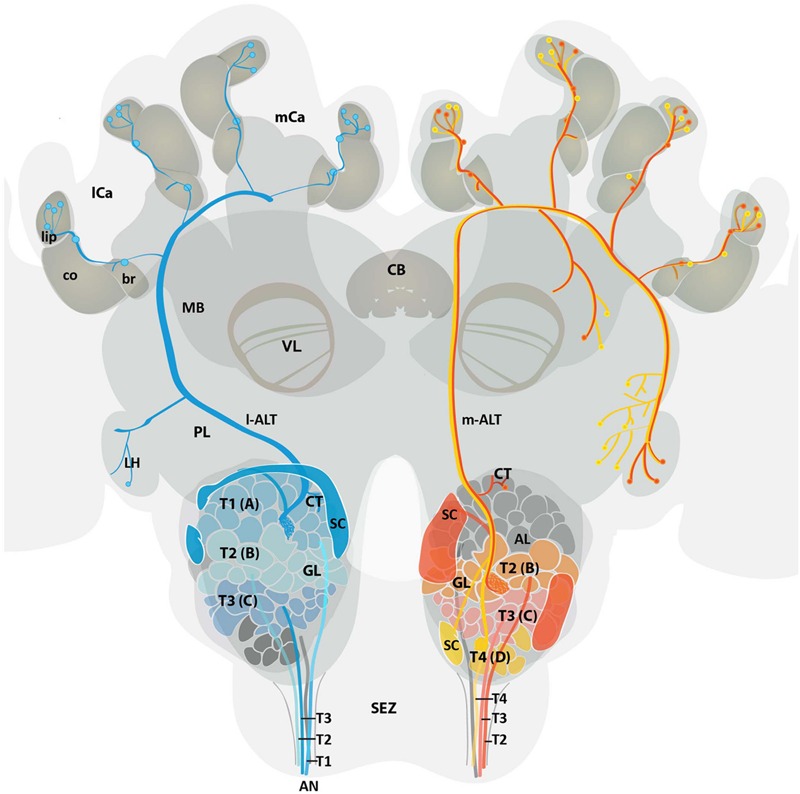
**Schematic overview of the medial and lateral antennal lobe tract neuronal pathways in the honeybee brain.** Overview displays the projection pattern of uniglomerular m-ALT (red and yellow) and l-ALT (blue) neurons in the honeybee brain. Four antennal tracts (T1, T2, T3, and T4) provide input in four glomerular cluster (T1, T2, T3, and T4) of the AL. Single glomeruli (GL) are referred to as A (T1 cluster), B (T2 cluster), C (T3 cluster), and D (T4 cluster) glomeruli. The l-ALT receives input from the T1–T3 cluster glomeruli, whereas the m-ALT gets input from T2–T4 cluster glomeruli. Somata of the l-ALT neurons are located in the dorsal, lateral, and anterio-lateral areas of the AL whereas m-ALT somata lie in the dorsal and ventro-lateral parts of the AL (SC, in blue and red, respectively). Both types of uPNs invade the MB calyces and the LH in a reverse manner. The m-ALT reaches the MB first and then projects to the LH, whereas the l-ALT courses in an opposite manner. Two types of m-ALTs exist. The sparse innervation type (red, input from T2 and T3 glomeruli), innervates only the LH whereas the broad innervation type (yellow, input from T4 glomeruli) invades the LPL outside the LH and the LH proper. Data modified from Abel et al. (2001); Kirschner et al. (2006) and current data. AL, antennal lobe; AN, antennal nerve; br, basal ring; CB, central body; CT, collaterals; co, collar; GL, glomeruli; l-ALT, lateral antennal lobe tract; PL, protocerebral lobe; lCa, lateral calyx; LH, lateral horn; m-ALT, medial antennal lobe tract; MB, mushroom body; mCa, medial calyx; SC, soma cluster; SEZ, subesophageal zone; T, tract; VL, vertical lobe of the MB.

By analyzing 12 single-cell stained ALT neurons, six m-ALT and six l-ALT cells, we were able to characterize in more detail similarities and differences of the neurons composing the dual olfactory pathway (**Figure 7**). Our data complements findings previously published by Abel et al. (2001), Müller et al. (2002), Kirschner et al. (2006). The m-ALT gets input from T2–T4 glomeruli whereas the l-ALT receives input from the T1–T3 glomeruli. All ALT neuron types display boutons in the basal ring, in or next to the collar, and lip region of the MB (compare **Figure 2**). Both m-ALT and l-ALT neurons innervate the core and the cortex of the ipsilateral MB lip. Inside the AL, neurons of both tracts send collaterals most likely onto the other tract (**Figure 4**). Only T4 m-ALT neurons send collaterals to the PL posterior to the MB. uPN boutons were found in the MB collar suggesting that the collar is a multimodal site like the basal ring and the lip region. Finally, our data support the conclusion that reciprocal synapses exist between PNs, octopaminergic, and GABAergic cells in the MB calyces.

## Author Contributions

HZ: conception and design, acquisition of data, analysis and interpretation of data, writing the article; DM: conception and design, acquisition of data, analysis and interpretation, revising the article; GM: acquisition of data; RM: conception and design, revising the article; JR: conception and design, analysis and interpretation of data, revising the article.

## Conflict of Interest Statement

The authors declare that the research was conducted in the absence of any commercial or financial relationships that could be construed as a potential conflict of interest.

## Footnotes

1 http://www.neurobiologie.fu-berlin.de/beebrain

## Abbreviations

AL: antennal lobe
ALT: antennal lobe tract
BAC: bilateral antennal lobe connecting neuron
br: basal ring
Ca: calyx
CB: central body
co: collar
d: dorsal
GABA: gamma-aminobutyric acid
GAD: glutaminacid decarboxylase
GL: glomeruli
IRT: inner ring tract
KC: Kenyon cell
l: lateral
lCa: lateral calyx
LH: lateral horn
LPL: lateral protocerebral lobe
m: medial
MB: mushroom body
mCa: medial calyx
OA: octopamine
ORN: olfactory receptor neuron
PCT: protocerebral-calycal tract
PL: protocerebral lobe
PN: projection neuron
SEZ: subesophageal zone
T: antennal lobe region
uPN: uniglomerular projection neuron
VL: vertical lobe
