# Supplementary material for: The Circuitry of Olfactory Projection Neurons in the Brain of the Honeybee, Apis mellifera
Source: Front Neuroanat. 2016 Sep 29;10:90. doi: 10.3389/fnana.2016.00090 (PMC5040750; doi:10.3389/fnana.2016.00090)
Supplement: Supplementary file 1 [file Supplementary_Data.PDF]

Quantitative morphology of Projection Neurons
of the lateral and median Antennal Lobe Tract

Cells

m- / l-ALT	glomerulus	AL sensory tract	Vol	Vol lat	Vol med	Length	Length lat	Length med
L1	B02-T2	T2	33630	11575	9541	11965	6439	5526
L2	A	T1	9999	5609	4390	9310	4920	4390
L3	A51-T1	T1	14130	8379	5751	9713	5206	4507
L4	A60-T1	T1	19115	11579	7536	8864	4984	3880
L5	A44-T1	T1	12514	6577	5937	8953	4166	4787
L6	A33-T1	T1	no	no	no	no	no	no
**M5**	**B05-T2**	**T2**	**62893**	**27763**	**35130**	**20261**	**10204**	**10057**
**M6**	**C**	**T3**	**42608**	**16334**	**26274**	**25896**	**13687**	**12209**
**M2**	**C73-T3**	**T3**	**22729**	**11626**	**11103**	**10279**	**5392**	**4887**
**M4**	**D08-T4**	**T4**	**30106**	**16775**	**13331**	**15915**	**8189**	**7726**
**M3**	**D02-T4**	**T4**	**25548**	**14592**	**10956**	**15201**	**8627**	**6574**
**M1**	**D06-T4**	**T4**	**71561**	**39303**	**32258**	**33922**	**18755**	**15167**

Distance to Centerline

m- / l-ALT	lat/med calyx	cell	>=0 | <0.05	>=0.05 | <0.1	>=0.1 | <0.15	>=0.15 | <0.2	>=0.2 | <0.25	>=0.25 | <0.3
L1	lat	B02	0	0	0	1	3	0
L2	lat	A	0	1	0	4	2	6
L3	lat	A51	0	2	3	2	5	8
L4	lat	A60	0	0	0	0	1	1
L1	med	B02	0	0	1	2	1	0
L2	med	A	1	4	3	3	4	5
L3	med	A51	2	0	1	1	6	10
L4	med	A60	0	0	0	0	0	0

APT			>=0 | <0.05	>=0.05 | <0.1	>=0.1 | <0.15	>=0.15 | <0.2	>=0.2 | <0.25	>=0.25 | <0.3
**M1**	**lat**	**D06**	**2**	**3**	**8**	**11**	**15**	**18**
**M2**	**lat**	**C73**	**0**	**3**	**1**	**1**	**1**	**5**
**M3**	**lat**	**D02**	**2**	**3**	**8**	**10**	**21**	**16**
**M4**	**lat**	**D08**	**1**	**8**	**8**	**15**	**16**	**21**
**M1**	**med**	**D06**	**1**	**3**	**8**	**10**	**18**	**26**
**M2**	**med**	**C73**	**0**	**1**	**0**	**1**	**2**	**5**
**M3**	**med**	**D02**	**2**	**7**	**10**	**8**	**12**	**17**
**M4**	**med**	**D08**	**1**	**0**	**5**	**5**	**11**	**14**

Boutons

whole calyx incl en passant	Blebs lat incl en passant	Blebs med incl en passant	sum lip	sum lat	sum med	Inner	Outer
469	260	209	222	115	107	66	156
391	206	185	314	169	145	109	205
479	258	221	327	176	151	214	113
319	176	143	166	94	72	40	126
221	113	108	208	100	108	124	84
145	90	55	126	80	46	67	59
1094	542	552	645	306	339	347	298
1288	645	643	831	392	439	451	380
409	228	181	324	181	143	132	192
795	420	375	616	332	284	219	397
673	401	272	513	293	220	231	282
1683	927	756	1100	483	617	737	363

Distance to Centerline

<table>
<tr><th>>=0.3 | <0.35</th><th>>=0.35 | <0.4</th><th>>=0.4 | <0.45</th><th>>=0.45 | <0.5</th><th>>=0.5 | <0.55</th><th>>=0.55 | <0.6</th><th>=0.6 | <0.6</th><th>=0.65 | <0.</th></tr>
<tr><td>2</td><td>5</td><td>1</td><td>0</td><td>7</td><td>7</td><td>10</td><td>14</td></tr>
<tr><td>10</td><td>9</td><td>18</td><td>14</td><td>12</td><td>13</td><td>13</td><td>13</td></tr>
<tr><td>9</td><td>8</td><td>9</td><td>13</td><td>11</td><td>16</td><td>14</td><td>16</td></tr>
<tr><td>1</td><td>4</td><td>2</td><td>3</td><td>2</td><td>5</td><td>8</td><td>11</td></tr>
<tr><td>1</td><td>4</td><td>4</td><td>7</td><td>8</td><td>2</td><td>6</td><td>15</td></tr>
<tr><td>10</td><td>5</td><td>16</td><td>13</td><td>11</td><td>13</td><td>11</td><td>7</td></tr>
<tr><td>8</td><td>11</td><td>12</td><td>8</td><td>7</td><td>11</td><td>11</td><td>9</td></tr>
<tr><td>1</td><td>3</td><td>2</td><td>3</td><td>2</td><td>6</td><td>4</td><td>6</td></tr>
</table>

<table>
<tr><th>>=0.3 | <0.35</th><th>>=0.35 | <0.4</th><th>>=0.4 | <0.45</th><th>>=0.45 | <0.5</th><th>>=0.5 | <0.55</th><th>>=0.55 | <0.6</th><th>=0.6 | <0.6</th><th>=0.65 | <0.</th></tr>
<tr><td>25</td><td>29</td><td>25</td><td>31</td><td>31</td><td>53</td><td>49</td><td>66</td></tr>
<tr><td>5</td><td>12</td><td>7</td><td>16</td><td>14</td><td>14</td><td>17</td><td>14</td></tr>
<tr><td>13</td><td>19</td><td>24</td><td>15</td><td>14</td><td>22</td><td>25</td><td>27</td></tr>
<tr><td>20</td><td>25</td><td>23</td><td>13</td><td>28</td><td>17</td><td>24</td><td>31</td></tr>
<tr><td>23</td><td>25</td><td>30</td><td>26</td><td>41</td><td>31</td><td>44</td><td>42</td></tr>
<tr><td>3</td><td>11</td><td>12</td><td>10</td><td>13</td><td>17</td><td>11</td><td>11</td></tr>
<tr><td>6</td><td>9</td><td>15</td><td>13</td><td>19</td><td>13</td><td>12</td><td>18</td></tr>
<tr><td>11</td><td>15</td><td>19</td><td>17</td><td>17</td><td>27</td><td>21</td><td>20</td></tr>
</table>

Boutons

m-anterior inner	m-anterior outer	m-posterior inner	m-posterior outer	m-inner	m-outer	m-anterior	m-posterior	l-anterior inner
17	39	17	34	34	73	56	51	22
24	55	24	42	48	97	79	66	29
46	32	50	23	96	55	78	73	56
2	33	6	31	8	64	35	37	14
33	19	29	27	62	46	52	56	10
23	16	7	0	30	16	39	7	18

95	70	89	85	184	155	165	174	143
111	110	103	115	214	225	221	218	54
31	25	41	46	72	71	56	87	29
54	99	44	87	98	186	153	131	68
55	42	33	90	88	132	97	123	78
198	69	215	135	413	204	267	350	227

Distance to Centerline

>=0.7 | <0.75	>=0.75 | <0.8	>=0.8 | <0.85	>=0.85 | <0.9	>=0.9 | <0.95	>=0.95 | <=1
12	13	25	9	5	1
7	13	15	10	7	2
11	14	9	19	7	0
8	12	8	15	11	2
7	16	14	9	10	0
7	9	8	8	5	2
10	9	11	12	9	3
8	6	16	9	5	1

>=0.7 | <0.75	>=0.75 | <0.8	>=0.8 | <0.85	>=0.85 | <0.9	>=0.9 | <0.95	>=0.95 | <=1
61	73	69	61	43	7
12	23	15	8	12	1
25	19	13	15	1	1
27	20	18	11	5	1
55	60	69	61	41	3
9	7	10	7	10	3
15	6	14	13	10	1
31	21	16	20	11	2

Boutons

l-anterior outer	l-posterior inner	l-posterior outer	l-inner	l-outer	l-anterior	l-posterior	LH Blebs	LH projections	PL projections
36	10	47	32	83	58	57	+	+	-
58	32	50	61	108	87	82	+	+	-
24	62	34	118	58	80	96	+	+	-
34	18	28	32	62	48	46	+	+	-
5	52	33	62	38	15	85	+	+	-
24	19	19	37	43	42	38	+	+	-
105	20	38	163	143	248	58	+	+	?
55	183	100	237	155	109	283	+	+	few
45	31	76	60	121	74	107	+	+	-
95	53	116	121	211	163	169	+	+	+
55	65	95	143	150	133	160	+	+	+
95	97	64	324	159	322	161	+	+	+

beneath lip projections	Collaterals near ALT	comments
-	+ +	
-	+	
-	+ possibly on l-ALT?	Blebs on axon before LH
-	+	
-	on m-ALT	structure on soma
-	possibly on m-ALT	
-	+ maybe on m-ALT	
few under lip	+ ++	
-	maybe on l-ALT	
+	-	
+	in the AL, spines?	
+	?	

Distance Mapping

	OA /PN			GABA/PN			GABA/ OA			Cell
	Length in µm	Contacts		Length in µm	Contacts		Length in µm	Contacts		
**preparation 1**	153	2								PN =T1 A33
triple-staining	239	0								
	377	0								
				293	21					
				361	42					
				377	12					
**preparation 2**				69	10					PN =T1 A44
double-staining				150	7					
				242	4					
				60	5					
				184	19					
				97	11					
				188	0					
				333	26					
				160	3					
				102	7					
				222	25					
				53	1					
**preparation 3**				149	5					WM 1
double-staining				40	0					
				675	8					
				127	2					
							45	1		
	112	0								
	127	1								
**preparation 4**							332	5		WM 2
double-staining							394	11		
							501	12		
							504	9		
	361	5								
**preparation 5**							600	10		WM 3
double-staining										
