# Supplementary material for: The Circuitry of Olfactory Projection Neurons in the Brain of the Honeybee, Apis mellifera
Source: Front Neuroanat. 2016 Sep 29;10:90. doi: 10.3389/fnana.2016.00090 (PMC5040750; doi:10.3389/fnana.2016.00090)
Supplement: Supplementary file 2 [file Supplementary_Figures.PDF]

Figure S1 Zwaka et al., 2016

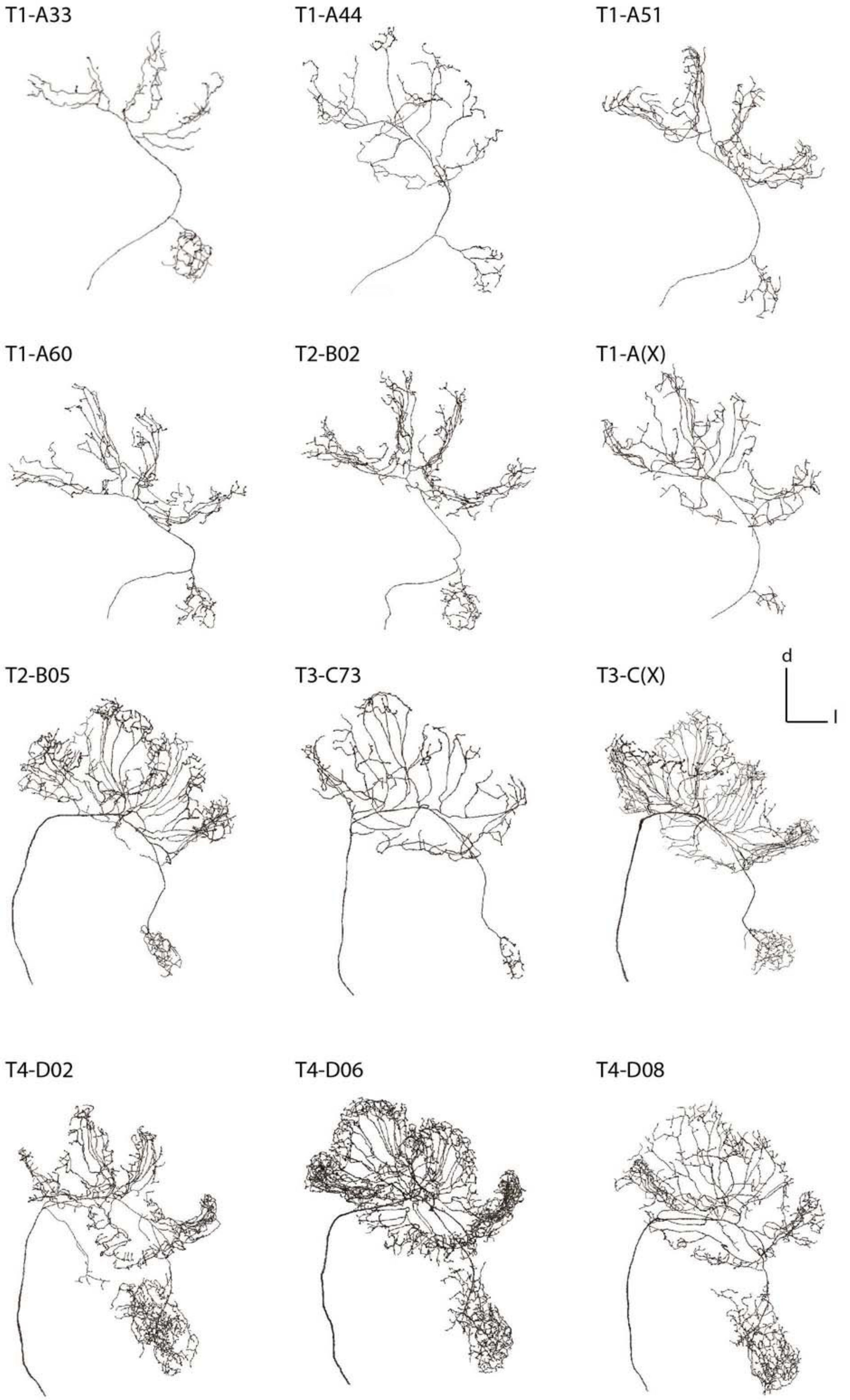

Figure S2 Zwaka et al., 2016

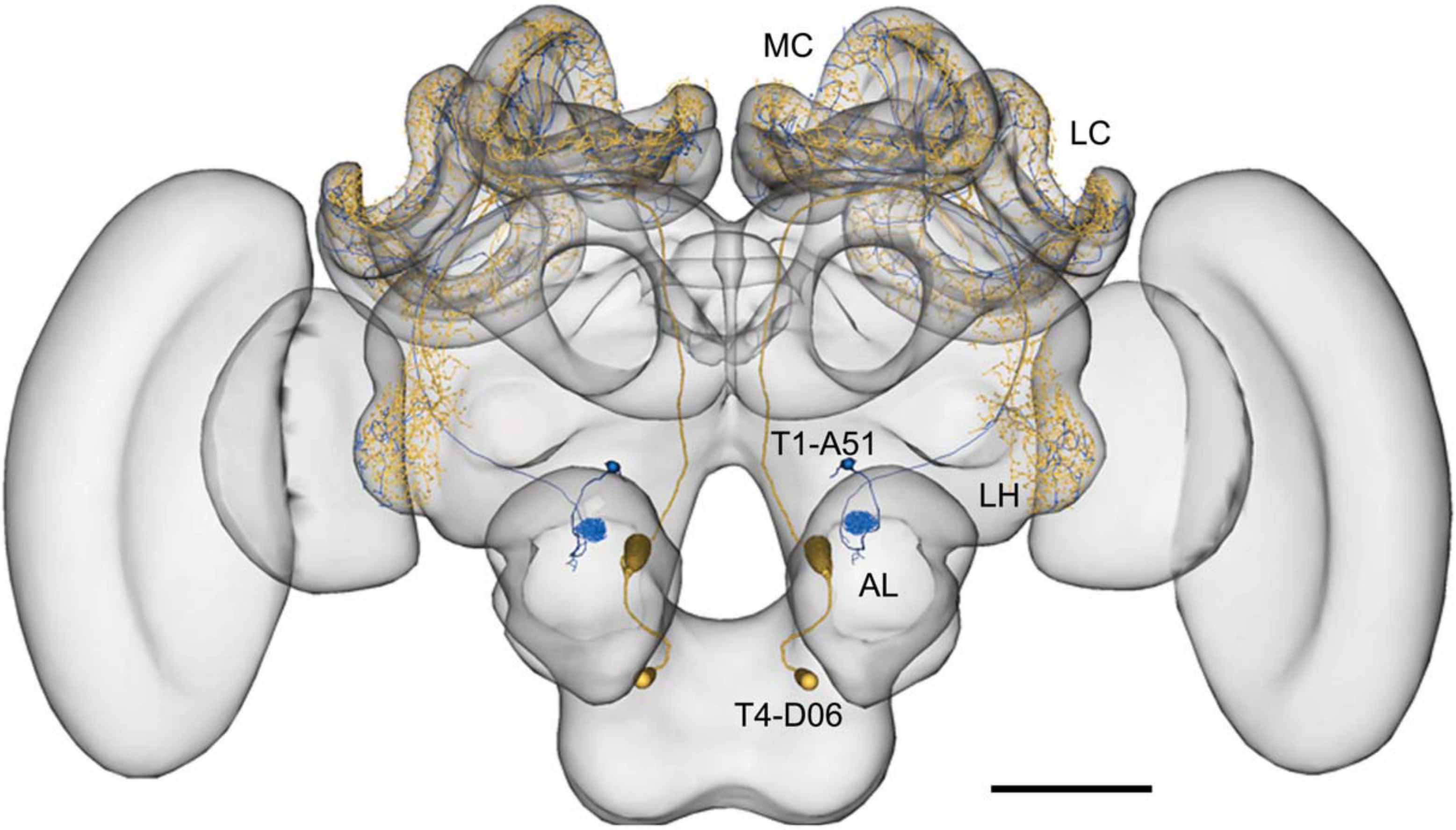

Figure S3 Zwaka et al., 2016

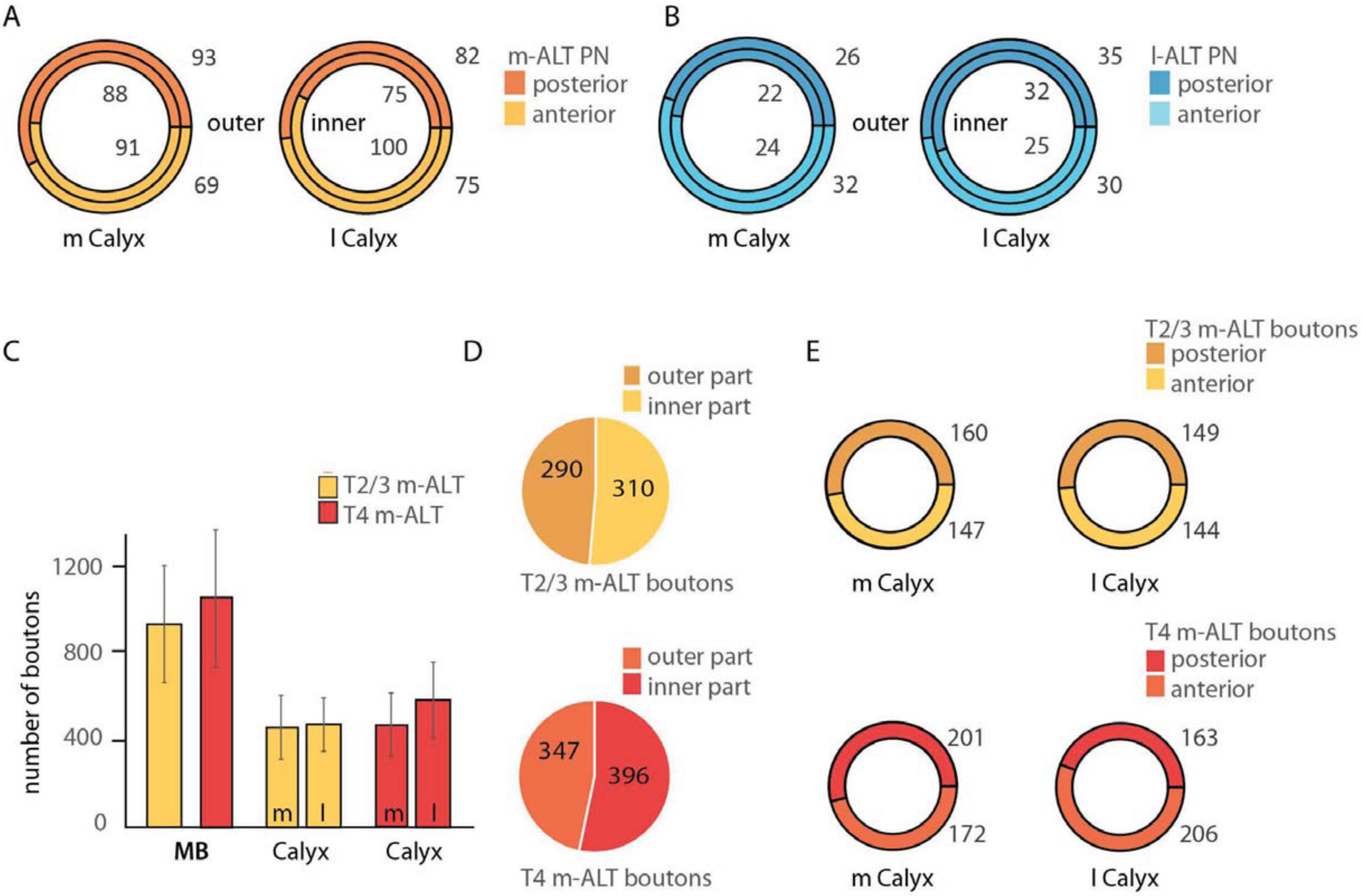

Figure S4 Zwaka et al., 2016

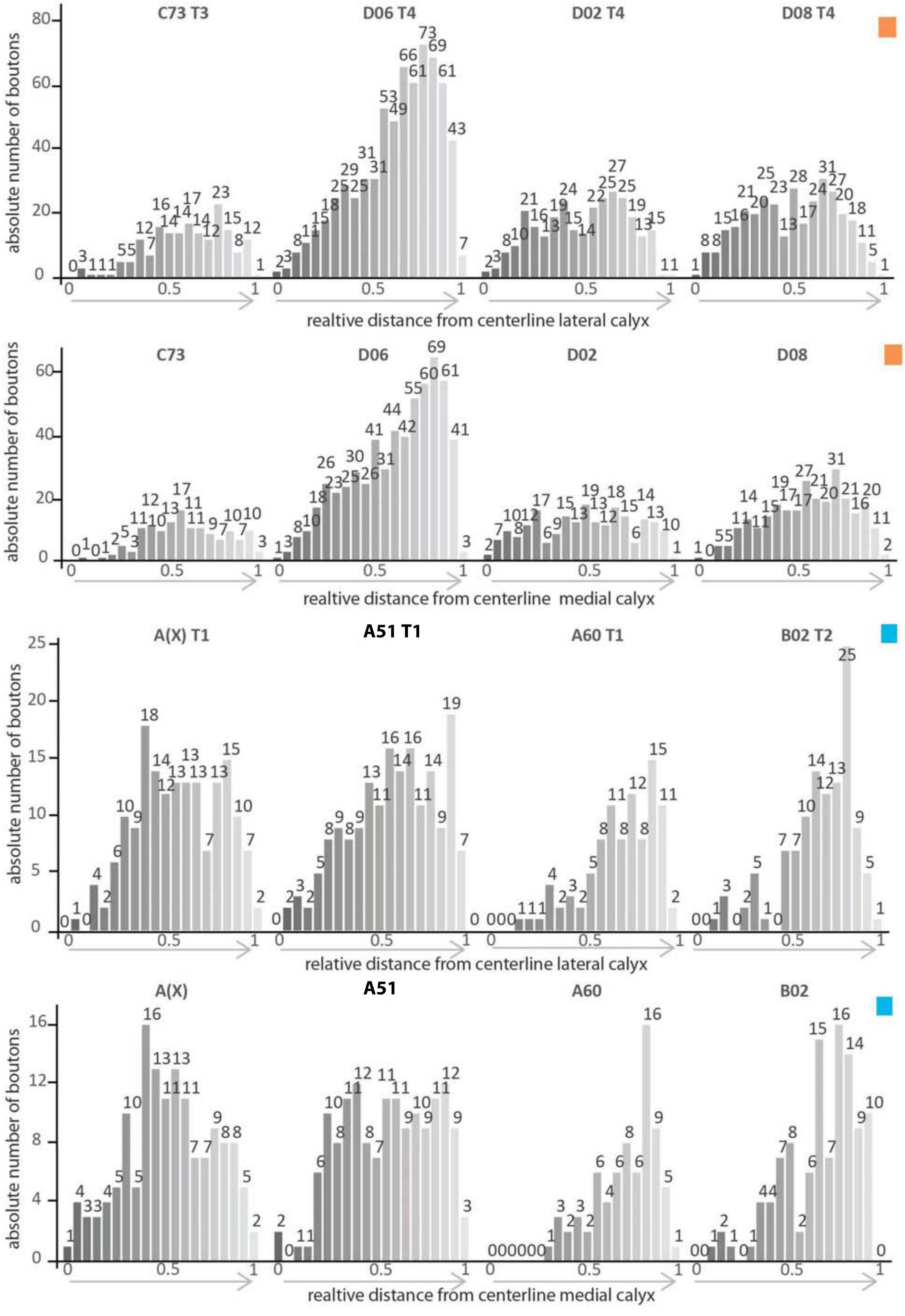

Figure S5 Zwaka et al., 2016

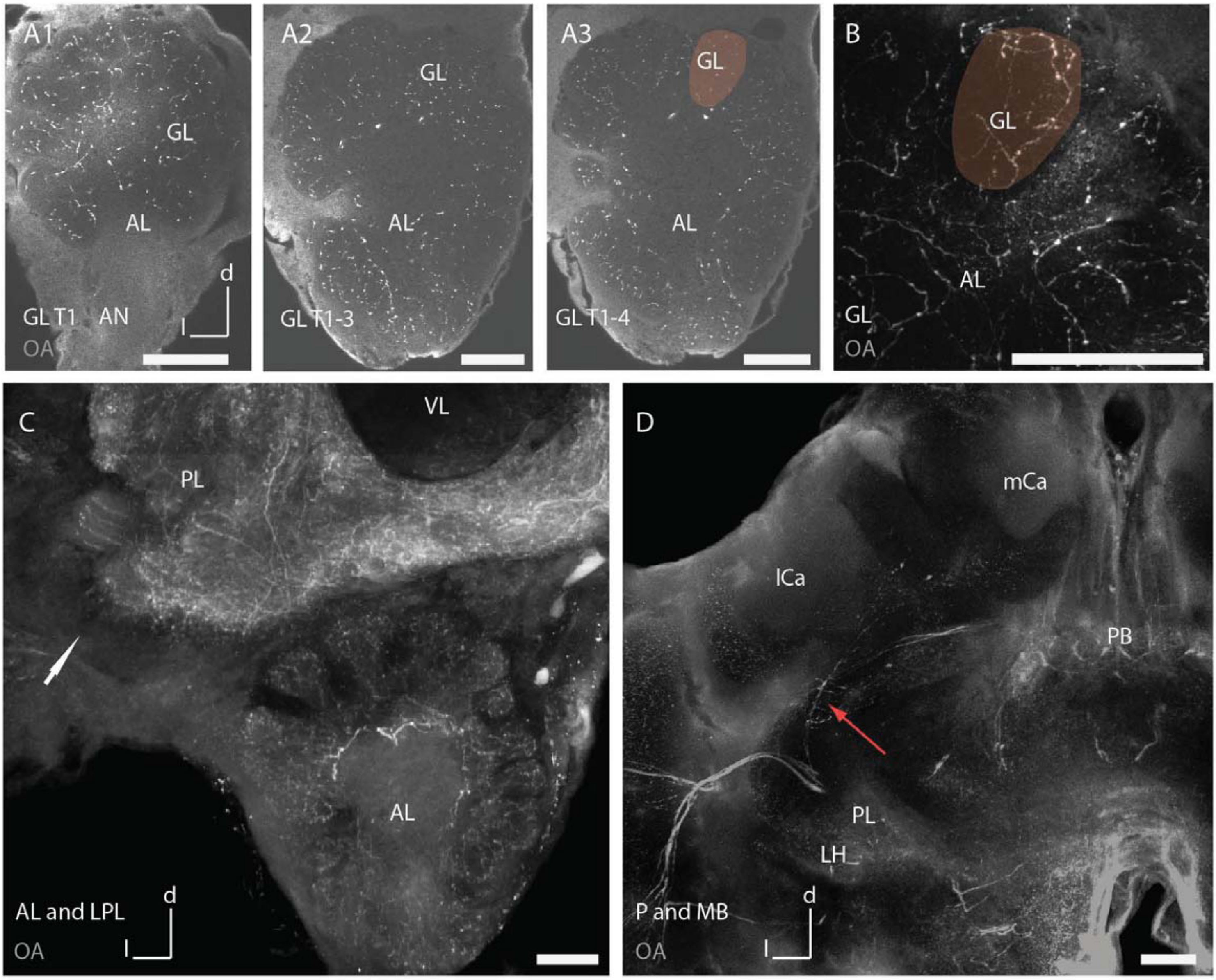

Figure S6 Zwaka et al., 2016

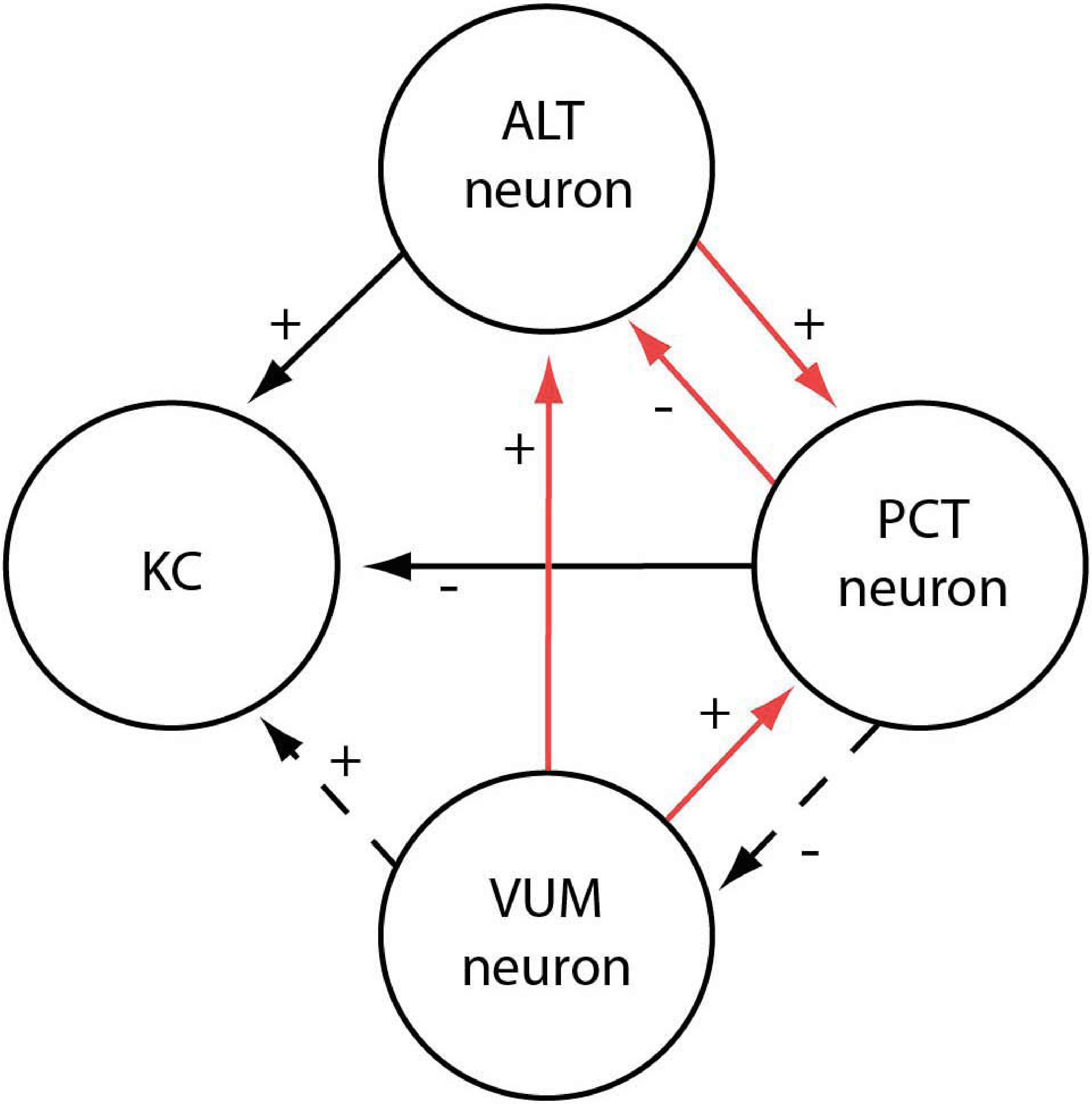

Legends for Supplementary Material Zwaka et al., 2016

Figure S1 Single neuron reconstruction of projection neurons of the medial (m-ALT) and lateral (l-ALT) antennal lobe tract in the honeybee brain

Summary of all reconstructed uniglomerular ALT neurons used in this work. The first two rows show l-ALT PNs with sparse innervation in the mushroom body calyces and the lateral horn. The third and fourth row depicts m-ALT PNs of two classes. T2/T3 m-ALT PNs with sparse and broad innervation of the MB calyces form terminals restricted to the lateral horn. T4 m-ALT PNs with exclusively broad domains in the MB calyces do occupy a large portion of the lateral protocerebral lobe. Letters and numbers give the antennal lobe region: T1-T4 and the innervated glomerulus: A-D, respectively.

Figure S2 An m-ALT and an l-ALT projection neuron registered to the standard Honeybee Standard Brain (HSB)

Two types of uniglomerular projection neurons were reconstructed and registered to the honeybee standard brain: A T4 m-ALT neuron (T4-D06, in yellow) and a T1 lALT (T1-A51, in blue). Note, the sparse innervation of l-ALT compared to the m-ALT PN in the mushroom body calyces (mCa, lCa) and the lateral horn (LH). For both neurons their respective mirror image was created and incorporated to the HSB to facilitate the comparison of their spatial relationships. AL, antennal lobe; lCa, lateral calyx; LH, lateral horn; mCa, medial calyx.

Figure S3 Comparison of bouton number in the mushroom body lip

(A, B) Comparison of the number of boutons in the posterior- (dark orange and blue) and anterior- (light orange and light blue), of the inner and outer part of the mushroom body lip of m-ALT (orange, N = 6) and l-ALT (blue, N = 6) neurons in percentage for the medial and lateral calyx. Numbers give the mean value of boutons in the compartments. **(C)** Comparison of T2/3 m-ALT neurons (yellow bars, N = 3) and T4 m-ALT neurons (red bars, N = 3) of the number of boutons (bar in red and yellow) in the whole MB calyces (MB, left part of the graph) and comparison of the number of boutons in the lateral versus the medial calyces (Calyx, right part of the graph). **(D)** Comparison of T2/3 m-ALT (orange, N = 3) and T4 m-ALT (blue, N = 3) neurons

of the number of boutons in the outer- (in orange) and inner- (in yellow and red) part for the lip in percentage displayed in a pie chart. Numbers give the mean value of boutons. N? T2/3 m-ALT = 3, inner: 310 +/- 94, outer: 290 +/- 54, T4 m-ALT = 3, inner: 396 +/- 171, outer: 347 +/- 34. **(E)** The total number of boutons in the posterior (darker color) and anterior (lighter color) parts of the lip of T2/3 m-ALT (yellow) and T4 m-ALT (red) neurons as a percentage, displayed as a pie chart for the medial (m Ca) and lateral calyx (l Ca). Numbers give the mean value of boutons in the compartments. NT2/3 m-ALT = 3, m-posterior: 160 +/- 38, m-anterior: 147+/-48, l-posterior: 149 +/- 68, l-anterior: 144 +/- 53, NT4 m-ALT = 3, m-posterior: 201 +/- 74, m-anterior: 172 +/- 50, l-posterior: 163 +/- 3, l-anterior: 206 +/- 59. l, lateral; m, medial; m-ALT, medial antennal lobe tract; MB, mushroom body. Error bars indicate SEM.

Figure S4 Distribution of uPN boutons in the mushroom body lip

A histogram shows the distribution of boutons in the lip with regard to their location between the centerline (0 = relative distance) and the surface (1 = relative distance) for single m-ALT and l-ALTs, respectively. Boutons are binned in 20 classes of equal width for relative distance in the lip (0.05 = relative distance). Bouton quantity is displayed as absolute number of boutons in the class. Four l-and four m-ALT neurons were analyzed and named according to their glomerular origin (A-D) and their participation to the respective antennal lobe tract (T1 - T4). L-ALT, orange; m-ALT, blue.

Figure S5 Octopamine-like immunoreactivity in the honeybee brain

(A1-A3) Frontal sections in different depths of the antennal lobe (AL) showing the innervation of all glomeruli by octopamine-like immunoreactive (OA-IR) fibers. The antennal nerve (AN) is void of staining. **(B)** Close-up of glomerular innervation by OA fibers. The reddish color marks one selected glomerulus (GL). **(C)** OA-IR distribution in the protocerebral lobe and the AL. The vertical lobe of the mushroom body is innervated only sparsely. Note, OA-IR fibers running in the anterior optic tubercle-to-lobula tract (a.o.t.t.) (Mobbs, 1982) projecting from the dorsal protocerebral lobe to the optical lobes (white arrow). **(D)** Frontal view reveals large OA-IR axons running in the l-ALT toward the calyces (red arrow). OA soma clusters are located at the medial sides of the ALs and close to the esophagus. The protocerebral bridge is innervated by several

crossing strands. Each calyx is innervated by OA fibers with arborizations in the lips, collar, and basal ring. Scale = 50 µm. AL, antennal lobe; AN, antennal nerve; CB, central body; GL, glomerulus; l-ALT, lateral antennal lobe tract; LH, lateral horn; PL, protocerebral lobe; lCa, lateral calyx; m-ALT, medial antennal lobe tract; mCa, medial calyx; PB, protocerebral bridge.

Figure S6 Putative synaptic connections in the lip of the honeybee mushroom body calyces. A connectivity map for neurons of the antennal lobe tract (ALT), the protocerebral-calycal tract (PCT) and the ventral unpaired median neuron (VUM). ALT-neurons receive inhibitory input (indicated by -) from PCT neurons and are presynaptically connected to PCT neurons and Kenyon cells (KCs) (indicated by +). PCT neurons inhibit KCs and form contacts to the VUM neuron (dashed line). The VUM neuron projects onto ALT- and PCT neurons and possible form contact to KCs. Red arrows show putative synaptic contacts that were indicated or reproduced in this study, black arrows indicate synaptic contacts found in other studies (Ganeshina and Menzel, 2001; Grünewald, 1999b; Sinakevitch et al., 2011), dashed arrows stand for hypothesized contacts. - :inhibitory input, +: excitatory input. Modified from (Ganeshina and Menzel, 2001).
